# Taste papilla cell differentiation requires the regulation of secretory protein production by ALK3-BMP signaling in the tongue mesenchyme

**DOI:** 10.1242/dev.201838

**Published:** 2023-09-25

**Authors:** Mohamed Ishan, Zhonghou Wang, Peng Zhao, Yao Yao, Steven L. Stice, Lance Wells, Yuji Mishina, Hong-Xiang Liu

**Affiliations:** ^1^ Regenerative Bioscience Center, Department of Animal and Dairy Science, College of Agricultural and Environmental Sciences, University of Georgia, Athens, GA 30602, USA; ^2^ Complex Carbohydrate Research Center, University of Georgia, Athens, GA 30602, USA; ^3^ Department of Biologic and Materials Sciences, School of Dentistry, University of Michigan, Ann Arbor, MI 48109, USA

**Keywords:** Taste papillae, Cell differentiation, Mesenchymal-epithelial interaction, ALK3-BMP signaling, Wnt/β-catenin signaling, Secretory proteins

## Abstract

Taste papillae are specialized organs, each of which comprises an epithelial wall hosting taste buds and a core of mesenchymal tissue. In the present study, we report that during early taste papilla development in mouse embryos, bone morphogenetic protein (BMP) signaling mediated by type 1 receptor ALK3 in the tongue mesenchyme is required for epithelial Wnt/β-catenin activity and taste papilla differentiation. Mesenchyme-specific knockout (*cKO*) of *Alk3* using *Wnt1-Cre* and *Sox10-Cre* resulted in an absence of taste papillae at E12.0. Biochemical and cell differentiation analyses demonstrated that mesenchymal ALK3-BMP signaling governed the production of previously unappreciated secretory proteins, i.e. it suppressed those that inhibit and facilitated those that promote taste papilla differentiation. Bulk RNA-sequencing analysis revealed many more differentially expressed genes (DEGs) in the tongue epithelium than in the mesenchyme in *Alk3 cKO* versus control. Moreover, we detected downregulated epithelial Wnt/β-catenin signaling and found that taste papilla development in the *Alk3 cKO* was rescued by the GSK3β inhibitor LiCl, but not by Wnt3a. Our findings demonstrate for the first time the requirement of tongue mesenchyme in taste papilla cell differentiation.

## Teaser

**Summary:** The first report indicating that tongue mesenchyme is required for taste papilla cell differentiation.

## INTRODUCTION

Taste papillae in the mammalian tongue are specialized organs comprising an epithelial wall that hosts taste buds and a core of mesenchymal tissue. Although structurally recognizable taste buds form later (∼E18.5 in mice; Barlow, 2015) than taste papillae (E14.5; Barlow, 2015; Krimm et al., 2015), taste bud cell progenitors are specified early. In mice, the tongue emerges as lingual swellings on the branchial arches at embryonic day (E) 11.0-11.5. The homogeneous epithelial cells in the primordial tongue express the pan-taste cell marker keratin 8 (Krt8) (Kramer et al., 2019; Mbiene and Roberts, 2003) and the developing taste papilla marker sonic hedgehog (Shh) (Kramer et al., 2019; Castillo-Azofeifa et al., 2017; Liu et al., 2004; Mistretta et al., 2003). While the swellings fuse to form a spatulate tongue at E12, taste papillae appear as epithelial thickenings (papilla placodes) on the dorsal surface, and the Krt8^+^Shh^+^ cells can rapidly differentiate into two groups: taste papilla (Krt8^+^Shh^high^) and inter-papilla (Krt8^−^Shh^low^) cells. The former gives rise to taste bud cells (Thirumangalathu et al., 2009; Gaillard et al., 2015; Kramer et al., 2019); the latter gives rise to basal epithelial cells that remain as a progenitor population for the cell renewal of mature taste buds throughout their lifetime (Thirumangalathu et al., 2009; Miura et al., 2014; Okubo et al., 2009; Kramer et al., 2019).

There has been a well-documented general concept that cell differentiation of epithelial appendages requires mesenchymal-epithelial interactions via molecular signaling (Santosh and Jones, 2014). Unlike many other epithelial appendages, of which the regulation of their cell differentiation by the surrounding mesenchyme is well-characterized (Sennett and Rendl, 2012; Chuong et al., 2000), research on taste papilla and bud cell differentiation has mainly focused on signaling molecules within the epithelium (Gaillard et al., 2017; Iwatsuki et al., 2007; Liu et al., 2013, 2004, 2009, 2008, 2012b; Mistretta et al., 2003). Less is known about the roles of the underlying tongue mesenchyme (Beites et al., 2009; Petersen et al., 2011; Prochazkova et al., 2017). Among the multiple molecular signaling pathways regulating taste papilla formation and epithelial cell differentiation, the effects of bone morphogenetic protein (BMP) pathway are profound (Beites et al., 2009; Ishan et al., 2020; Zhou et al., 2006). However, the details are unclear, including the identity of the BMP receptor(s) involved in the signaling, the tissue compartments/cell types involved and the interactions with signaling in the epithelium.

In this study, we report that neural crest-derived mesenchyme-specific conditional knockout of type I BMP receptor *Alk3* (*Alk3 cKO*), but not *Alk2 cKO*, resulted in a complete loss of taste papilla placodes (i.e. taste cell progenitors). In combination with RNA sequencing, liquid chromatography-mass spectrometry (LC-MS) and cell differentiation analyses using tongue organ cultures, we found that *Alk3 cKO* mesenchyme secretes proteins ranging from 10-100 kDa that suppress the taste papilla cell differentiation through suppression of Wnt/β-catenin signaling activity. Together, our data demonstrate that BMP signaling mediated by ALK3 (hereafter ALK3-BMP) in neural crest-derived mesenchyme is essential for the differentiation of taste papilla cells through its suppression of the production of previously unappreciated mesenchymal secretory proteins and its promotion of epithelial Wnt/β-catenin signaling activity.

## RESULTS

### BMP signaling is active during early taste papilla differentiation, with the highest *Alk3* expression among type I receptors

**Fig. 1.**
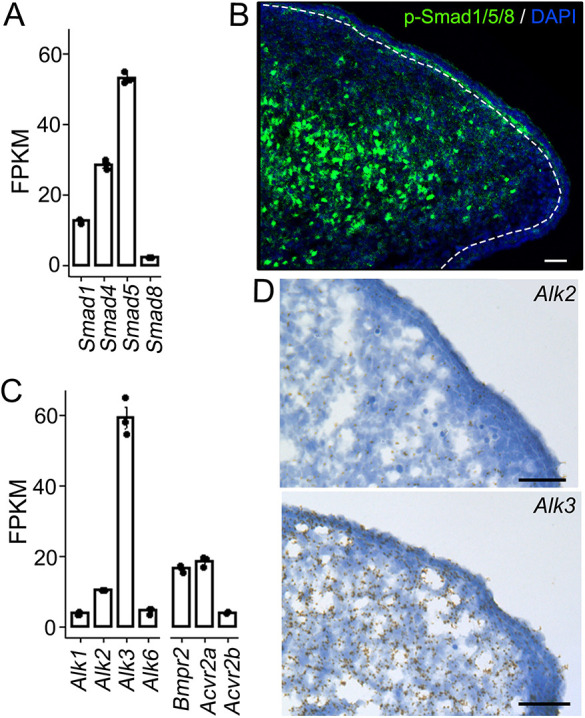
**The BMP signaling pathway is intact and active in E12.0-E12.5 mouse tongue tissues.** (A) Histogram (mean±s.d.; *n*=3) presenting the fragments per kilobase of transcript per million mapped reads (FPKM) values of BMP signaling molecules Smad1, Smad4, Smad5 and Smad8 in the tongue mesenchyme at E12.5. (B) A representative image (single-plane laser scanning confocal) of a sagittal tongue section at E12.5. The section was immunostained for the BMP signaling transcription factor p-Smad1/5/8 (green) and counterstained with DAPI (blue). Scale bar: 50 μm. (C) A histogram (mean±s.d.; *n*=3) presenting the expression level of BMP receptors as FPKM values in the tongue mesenchyme at E12.5. (D) Representative light microscopy images of E12.5 tongue sections. RNAscope *in situ* hybridization was performed using an antisense probe for *Alk2* or *Alk3* (brown dots). Sections were counterstained with 50% Hematoxylin to show cell nuclei (blue). Scale bars: 50 µm.

Phosphorylation (p) of Smad1/5/8 is a crucial step and reliable indicator of BMP signaling activity (Bragdon et al., 2011; Wang et al., 2014; Massagué et al., 2005; Retting et al., 2009). Transcripts of *Smad1*, *Smad4*, *Smad5* and *Smad8* were detected in the tongue mesenchyme at E12.5 (Fig. 1A), a stage when the tongue epithelial cells differentiate into two groups (Kramer et al., 2019; Okubo et al., 2006; Nakayama et al., 2008; Liu et al., 2004): Shh^high^Prox1^+^ taste cell progenitors in taste papilla placodes; and Shh^low^Prox1^−^ non-gustatory cells between papillae. At this stage, p-Smad1/5/8^+^ cells were abundantly distributed in the tongue mesenchyme and epithelium (Fig. 1B).

To determine the role of the BMP signaling pathway in taste organogenesis, we used transcriptomic analyses, which revealed that among the four type-I BMP receptors, the *Alk3* transcripts level was significantly higher than the other three (*Alk1*, *Alk2*, and *Alk6*) in the tongue mesenchyme (Fig. 1C) in the order of *Alk3*≫*Alk2*>*Alk6*≈*Alk1*. RNAscope *in situ* hybridization data further confirmed a significantly higher level of *Alk3* RNA expression than that of *Alk2* (Fig. 1D).

### Mesenchyme-specific knockout of *Alk3*, but not *Alk2*, leads to an absence of taste papillae in early embryos

**Fig. 2.**
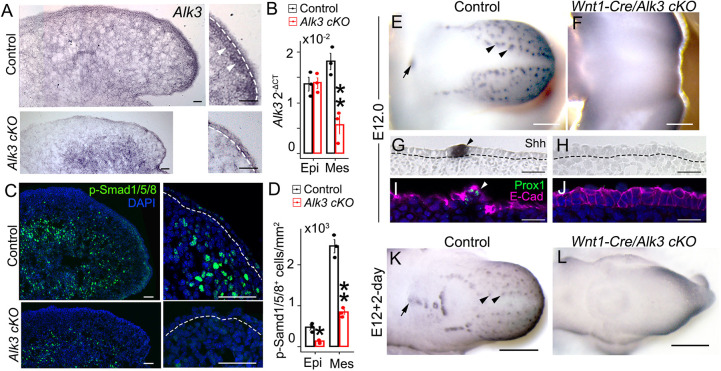
**Mesenchyme-specific *Wnt1-Cre/Alk3 cKO* results in a complete loss of taste papillae.** (A) Light microscopy images of sagittal tongue sections of E12.0 *Cre^−^/Alk3^fx/fx^* (control) and *Wnt1-Cre/Alk3 cKO* (*Alk3 cKO*) mice. *In situ* hybridization was performed using an antisense probe for *Alk3* (purple). White dashed lines separate the tongue epithelium from the underlying mesenchyme. Arrowheads indicate the mesenchyme underneath the epithelium. Scale bars: 100 µm. (B) A histogram (mean±s.d.; *n*=3) presenting the 2^−ΔCT^ values of *Alk3* gene transcripts in the tongue epithelium (Epi) and mesenchyme (Mes) of *Cre^−^/Alk3^fx/fx^* (control) and *Wnt1-Cre/Alk3 cKO* (*Alk3 cKO*) mouse embryos at E12.0. **P≤0.01 compared with control using two-way ANOVA followed by Fisher's least significant difference (LSD) analysis. (C) Single-plane laser-scanning confocal images of sagittal tongue sections that were immunostained for p-Smad1/5/8 (green) in control and *Wnt1-Cre/Alk3 cKO* mice. The right column shows the anterior tongue region at a higher magnification. Dashed lines separate the tongue epithelium from the underlying mesenchyme. Scale bars: 50 μm. (D) A histogram (mean±s.d.; *n*=3) presenting the number of p-Smad1/5/8^+^ cells per mm^2^ in the tongue epithelium (Epi) and mesenchyme (Mes). *P≤0.05, **P≤0.01 compared with controls using two-way ANOVA followed by Fisher's LSD analysis. (E-H) Representative light microscopy images of E12.0 tongues immunostained for the developing taste papilla marker sonic hedgehog (Shh) (blue). Tongues are from control (E,G) and *Wnt1-Cre/Alk3 cKO* (F,H). (G,H) Images of sagittal tongue sections. Dashed lines separate the tongue epithelium from the underlying mesenchyme. Arrowheads and the arrow indicate Shh^+^ fungiform and circumvallate papilla placodes, respectively. (I,J) Single-plane laser-scanning confocal images of sagittal tongue sections that were immunostained for the taste papilla marker Prox1 (green) and epithelial cell marker E-cadherin (E-cad). Tongues are from control (I) and *Wnt1-Cre/Alk3 cKO* (J) mice. The arrowhead indicates a Prox1^+^ fungiform papilla. Scale bars: 50 μm. (K,L) Representative light microscopy images of E12+2-day tongue cultures from control and *Wnt1-Cre/Alk3 cKO* mice immunostained for Shh. Tongues were cultured with standard medium. Arrowheads and the arrow indicate Shh^high^ fungiform and circumvallate papilla placodes, respectively. Scale bars: 200 µm.

To define the role of ALK3-BMP signaling in the tongue mesenchyme, a neural crest (NC)-derived mesenchyme-specific knockout of *Alk3* (*Alk3 cKO*) was generated using *Wnt1-Cre* (Jiang et al., 2000), which marks the NC cell lineage in the tongue mesenchyme extensively (Thirumangalathu et al., 2009; Ishan et al., 2021; Yu et al., 2020; Liu et al., 2012a). In *Wnt1-Cre/Alk3 cKO* tongues, *Alk3* transcripts were significantly reduced in the mesenchyme (Fig. 2A, *P*<0.05 in Fig. 2B), especially in the mesenchyme immediately under the epithelium, compared with control (arrowheads in Fig. 2A). In accordance with the reduction in *Alk3* transcripts, p-Smad1/5/8^+^ cells were seen in a significantly lower number in the tongue mesenchyme of E12.0 *Wnt1-Cre/Alk3 cKO* tongue compared with *Cre^−^* littermates (Fig. 2C, *P*<0.05 in Fig. 2D). The difference was especially obvious in the mesenchymal layer immediately under the epithelium (Fig. 2C).

A dramatic phenotype of mesenchyme-specific deletion of ALK3-BMP signaling is the complete loss of taste papillae (i.e. taste cell progenitors) in E12.0 *Wnt1-Cre/Alk3 cKO* (Fig. 2F) compared with the *Cre^−^* littermate controls (Fig. 2E). The papilla loss was confirmed by the lack of thickening of the tongue epithelium marked by a well-documented and reliable marker (Beites et al., 2009; Castillo et al., 2014; Ishan et al., 2020; Iwatsuki et al., 2007; Jung et al., 1999; Liu et al., 2008, 2004; Mistretta et al., 2003; Zhou et al., 2006; Hall et al., 1999, 2003) – intense Shh immunosignals (Fig. 2H versus G) and Prox1 (Fig. 2J versus I) expression on serial sections of *Alk3 cKO* tongues (Fig. 2H,J). In addition, a truncation of the tip of tongue/mandible was observed in *Wnt1-Cre/Alk3 cKO* mice (Fig. 2F). Of note, mesenchyme-specific knockout of another BMP receptor, *Alk2*, did not lead to an absence of taste papillae (Fig. S1); instead, well-developed and stereotypically located fungiform (arrowheads in Fig. S1) and circumvallate (the arrow in Fig. S1) papilla placodes were seen in the *Wnt1-Cre/Alk2 cKO*, similar to the *Cre^−^* littermates (Fig. S1).

Owing to the embryonic lethality of *Alk3* c*KO* driven by *Wnt1-Cre* (sudden death caused by heart failure at E12.5 or later) (Stottmann et al., 2004), close attention was paid to the condition of all embryos at collection (E12 or younger) to ensure the rigorous heart beat and blood circulation. Cell viability was evaluated using cell proliferation and apoptosis markers, including Ki67^+^ (pan proliferation), BrdU^+^ (S-phase), p-H3^+^ (M-phase) and cleaved (c)-Caspase3^+^ (c-Cas3, apoptosis). No changes in cell proliferation and apoptosis were detected in *Wnt1-Cre/Alk3 cKO* tongues compared with that of *Cre^−^* littermates (Fig. S2).

To evaluate the developmental course of taste papilla absence in the mesenchymal *Alk3 cKO*, phenotypes were analyzed at earlier stages (E10.5-E11.5) in embryos and at later stages in developed tongue organs in the 2-day cultures started at E12.0. Alterations of tongue development were not obvious in *Alk3 cKO* until E11.5, at which point the lateral tongue swellings were smaller, at an 80% occurrence rate in *Alk3 cKO* compared with littermate controls with the same somite numbers (Fig. S3). The four branchial arches (E10.5) and early tongue swellings (E11.0) developed in *Wnt1-Cre/Alk3 cKO* mice similarly to *Cre^−^* littermates (Fig. S3). After a 2-day extension of E12 tongue development in culture, taste papillae did not form in *Wnt1-Cre/Alk3 cKO* (Fig. 2L). The cultured *Alk3 cKO* tongues that were free from the restraint of mandible displayed apparent outgrowth and a pointed tip (Fig. 2L), in contrast to the truncated tip of E12 *Alk3 cKO* tongue (Fig. 2F).

To address whether an elevated level of ALK3-BMP signaling in the tongue mesenchyme alters taste papilla differentiation, *Wnt1-Cre* mediated constitutively activated (ca) *Alk3* (*Wnt1-Cre/caAlk3*) was used. No obvious changes in the taste papilla and taste bud development were found (Fig. S4). Similar to the *Cre^−^* littermate controls, fungiform and circumvallate papillae (E12.5 and E14.5) and taste buds (postnatal day 21) developed in the *Wnt1-Cre/caAlk3* mice (Fig. S4).

### Mesenchymal *Alk3 cKO* results in taste papilla absence through tongue mesenchyme-produced proteins

**Fig. 3.**
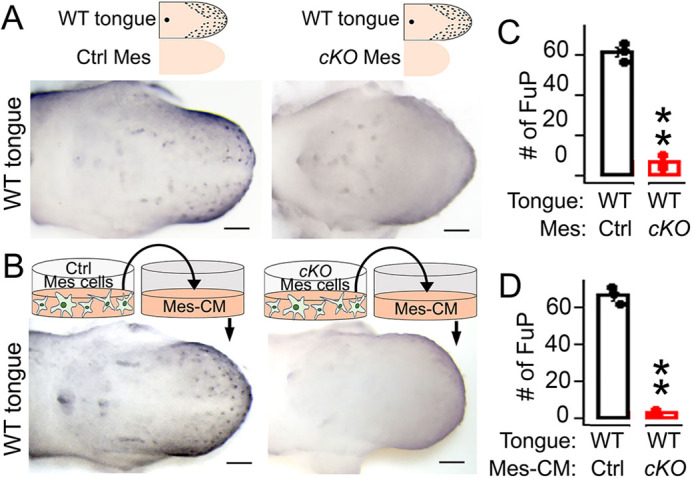
**Taste papilla differentiation is suppressed by *Wnt1-Cre/Alk3 cKO* tongue mesenchyme and its conditioned medium in E12.0+2-day wild-type tongue cultures.** (A,B) Representative light microscopy images of E12.0+2-day tongue cultures from wild-type mice. Cultures were either co-cultured with tongue mesenchyme (Mes) (A) or fed with mesenchyme-conditioned medium (Mes-CM) (B) and immunostained for Shh (blue). Ctrl, *Cre^−^/Alk3^fx/fx^*; cKO, *Wnt1-Cre/Alk3 cKO*. The schematic diagrams illustrate how the experiment was set up. Scale bars: 200 µm. (C,D) Histograms (mean±s.d.; *n*=3) presenting the number of Shh^high^ fungiform papillae (FuP) in cultures that were either co-cultured with tongue mesenchyme (Mes) or fed with mesenchyme-conditioned medium (Mes-CM) from control or *cKO* mice. **P≤0.01 compared with *Cre^−^/Alk3^fx/fx^* littermate control using two-way ANOVA followed by Fisher's least significant difference (LSD) analysis.

Tissue-tissue interactions may be through direct contact (Cutler and Gremski, 1991) and/or paracrine signals (Thesleff et al., 1988; Sagmeister et al., 2008; Horowitz and Thannickal, 2006; Chapman, 2011; Prochazkova et al., 2017). To address how *Alk3 cKO* tongue mesenchyme causes taste papilla absence in the epithelium, co-cultures without direct contact were used. Given that at E12 tongue epithelial cells undergo rapid differentiation, culturing the whole tongue is essential to preserve the integrity of the tongue epithelium. Therefore, tongue mesenchymal tissue of E12.0 *Wnt1-Cre/Alk3 cKO* or *Cre^−^* littermates was placed adjacent to, but not in direct contact with, E12.0 wild-type tongues in the 2-day cultures (Fig. 3A,B). In the wild-type tongues cultured with *Wnt1-Cre/Alk3 cKO* tongue mesenchyme, Shh^high^ taste papillae were significantly reduced in number and less intensely marked compared with those co-cultured with control tongue mesenchyme (Fig. 3A, *P*<0.01 in Fig. 3C). These data suggest that the tongue mesenchyme interacts with the epithelium in a paracrine manner. To further confirm this idea, we tested the effects of medium from tongue mesenchymal cell cultures (mesenchyme-conditioned medium – Mes-CM). We found that the morphology and proliferating rate of the tongue mesenchymal cells were similar in *Alk3 cKO* and control groups (Fig. S5). Mes-CM from *Wnt1-Cre/Alk3 cKO* tongues potently inhibited taste papilla differentiation in wild-type tongue cultures compared with control group (Fig. 3B, *P*<0.01 in Fig. 3D), which confirms the involvement of paracrine factors in the mesenchymal-epithelial interactions.

**Fig. 4.**
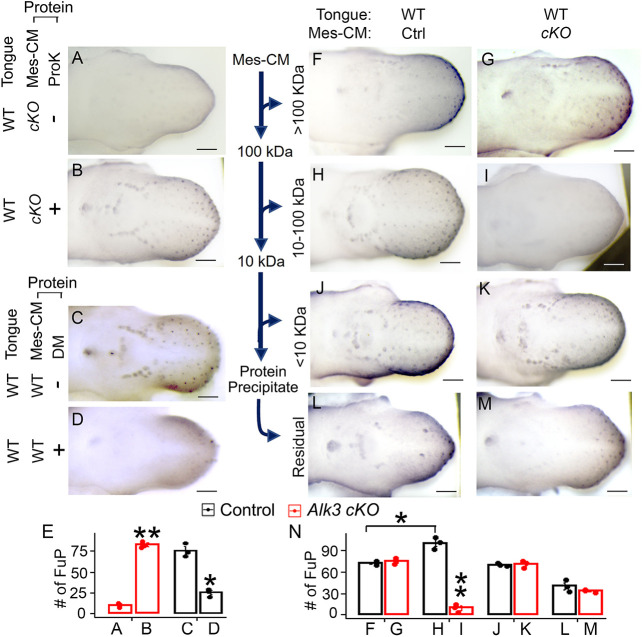
**Proteins from *Alk3 cKO* tongue mesenchyme-conditioned medium inhibit taste papilla differentiation.** (A-D,F-M) Representative light microscopy images of E12.0+2-day wild-type tongues cultures immunostained for the taste papilla marker Shh (blue). Proteins from mesenchyme-conditioned medium (Mes-CM) (A-D,F-K) or protein-free residual solution (L,M) were added to the culture medium. Ctrl, *Cre^−^/Alk3^fx/fx^* (F,H,J,L); *cKO*, *Wnt1-Cre/Alk3 cKO* (A,B,G,I,K,M); ProK, proteinase K; DM, dorsomorphin. Scale bars: 200 µm. (E,N) Histograms (mean±s.d.; *n*=3) presenting the number of Shh^high^ fungiform papillae (FuP) in the tongue cultures under different experimental conditions shown in A-D,F-M. *P≤0.05, **P≤0.01 compared with the littermate controls using two-way ANOVA followed by Fisher's LSD analysis.

To define the tongue mesenchyme-produced factors that impact taste papilla differentiation, proteins were extracted from Mes-CM; those from *Wnt1-Cre/Alk3 cKO* tongues showed an almost complete suppression of taste papilla formation (Fig. 4A,E). Proteinase K (ProK) pretreatment efficiently digested the extracted proteins from Mes-CM (Fig. S6) and eliminated the inhibition by proteins from *Alk3 cKO* tongue mesenchyme (Fig. 4B,E). Furthermore, in the E12+2-day wild-type tongue cultures, proteins extracted from the conditioned medium of mesenchymal cells treated with the p-Smad1/5/8 inhibitor dorsomorphin mimicked the taste papilla absence observed in *Alk3 cKO in vivo* (Fig. 4D versus C, *P*<0.05 in Figs 4E, 2F). The effects of mesenchyme-produced proteins were further analyzed using filtered protein fragments at different molecular weights. Administration of 10-100 kDa proteins from the control group to wild-type tongue cultures resulted in an increase in taste papilla number (Fig. 4H versus F,J,L, *P*<0.05 in Fig. 4N), whereas the 10-100 kDa proteins from *Alk3 cKO* lead to an absence of taste papillae (Fig. 4I versus G,K,M, *P*<0.01 in Fig. 4N), which mimics the *in vivo Alk3 cKO* phenotype (Fig. 2F). In contrast, >100 kDa or <10 kDa Mes-CM proteins, or protein-free residual solution did not result in a difference in taste papillae between *Wnt1-Cre/Alk3 cKO* and control groups (Fig. 4G versus F, K versus J, M versus L, *P*>0.05 in Fig. 4N).

### Mesenchymal *Alk3 cKO* leads to a downregulation of epithelial Wnt/β-catenin signaling

**Fig. 5.**
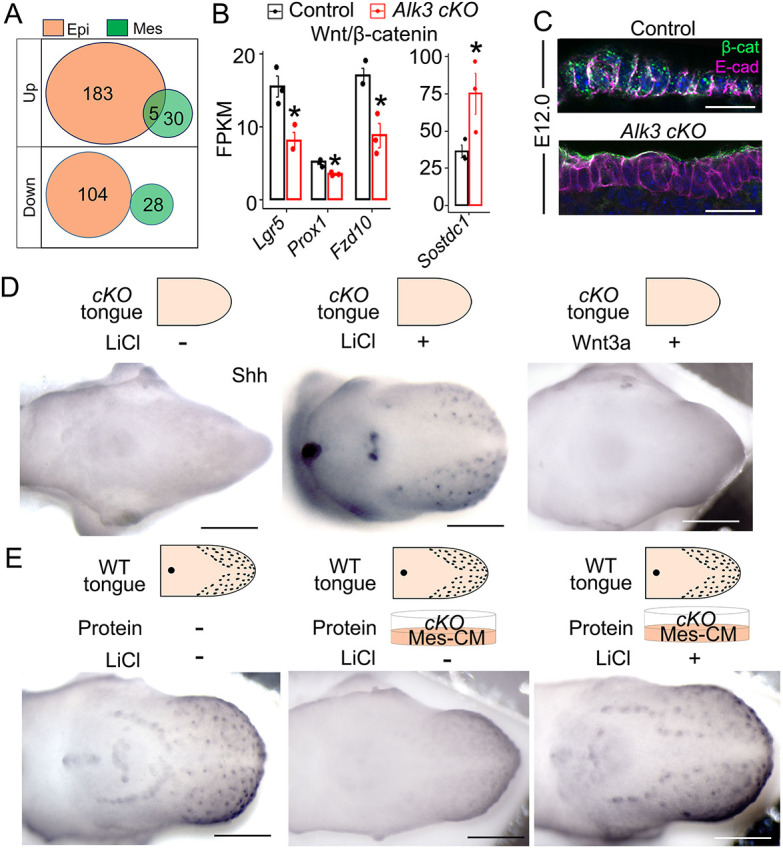
**Mesenchymal *Alk3 cKO* leads to the downregulation of Wnt/β-catenin signaling in the tongue epithelium and activating the pathway rescues taste papilla development.** (A) Venn diagram to show the number of differentially expressed genes (DEGs) in the tongue epithelium (Epi) and mesenchyme (Mes) of *Wnt1-Cre/Alk3 cKO* versus littermate control. Up, upregulated DEGs; Down, downregulated DEGs. (B) Histograms (mean±s.d.; *n*=3) presenting the FPKM values of Wnt/β-catenin signaling components in E12.0 tongue epithelium. Control, *Cre^−^/Alk3^fx/fx^*; *Alk3 cKO*, *Wnt1-Cre/Alk3 cKO*. *P≤0.05 (adjusted *P*-value); DESeq2 statistical analysis based on read counts. (C) Single-plane laser scanning confocal images of sagittal tongue sections that were immunostained for β-catenin (β-cat, green) and E-cadherin (E-cad, magenta). Tongues are from *Cre^−^/Alk3^fx/fx^* (control) and *Wnt1-Cre/Alk3 cKO* (*Alk3 cKO*) mice. Scale bars: 25 μm. (D,E) Representative light microscopy images of E12+2-day tongue cultures from *Wnt1-Cre/Alk3 cKO* (*cKO* in D) or wild-type (E) mice. Tongue cultures were treated with 5 mM LiCl or 20% Wnt3a conditioned medium to activate Wnt/β-catenin signaling and immunostained for Shh (blue). The schematic diagrams show how the experiment was set up. Mes-CM, mesenchyme-conditioned medium. Scale bars: 200 µm.

To unravel the molecular basis of the potent suppression of *Alk3 cKO* on taste papilla differentiation, RNA-sequencing analyses were performed on the separated tongue epithelium and mesenchyme in E12.0 *Wnt1-Cre/Alk3 cKO* versus littermate control (Table S1). Interestingly, many more differentially expressed genes (DEGs) were detected in the epithelium than in the mesenchyme (|FC|>1, *P*<0.05, FDR *q*<0.05) (Fig. 5A). Among the total 350 DEGs, 287 genes (183 upregulated and 104 downregulated) were detected in the epithelium, and 58 genes (30 upregulated and 28 downregulated) in the mesenchyme, and 5 genes were upregulated in both the epithelium and mesenchyme (Fig. 5A, Tables S2-S5).

Further analyses indicated a downregulation of Wnt/β-catenin pathway in the tongue epithelium of *Wnt1-Cre/Alk3 cKO* versus control. The epithelial Wnt/β-catenin-related DEGs include the downregulated positive regulators *Lgr5*, *Prox1* and *Fzd10*, and the upregulated inhibitor *Sostdc1* (Fig. 5B). The decreased Wnt/β-catenin activity in *Alk3 cKO* tongue epithelium was confirmed by the lack of nuclear accumulation of β-catenin that was apparent in the control, especially in taste papilla cells (Fig. 5C).

To test whether the downregulation of Wnt/β-catenin signaling is the main cause of taste papilla absence in mesenchymal *Alk3 cKO*, Wnt/β-catenin activators LiCl (Clément-Lacroix et al., 2005; Iwatsuki et al., 2007) or Wnt3a were added to the culture medium. The control group tongues depicted an increase in taste papilla number in the E12+2-day cultures with LiCl and Wnt3a (Fig. S7). However, taste papilla development in *Wnt1-Cre/Alk3 cKO* tongue cultures was rescued only when treated with LiCl but not with Wnt3a (Fig. 5D). Moreover, the administration of LiCl also rescued taste papilla loss in *Alk3 cKO* tongue Mes-CM protein-treated wild-type tongue cultures (Fig. 5E).

### Mesenchymal *Alk3 cKO* driven by *Sox10-Cre* mimics the phenotypic alterations seen in *Wnt1-Cre/Alk3 cKO*

**Fig. 6.**
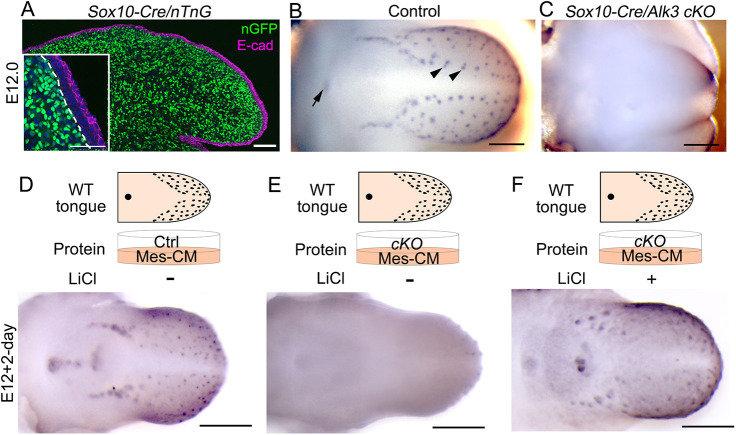
***Sox10-Cre/Alk3 cKO* depicts the taste papilla loss observed in *Wnt1-Cre/Alk3 cKO* mice.** (A) Single-plane laser-scanning confocal images of a sagittal tongue section from an E12.0 *Sox10-Cre/nTnG* mouse. The tongue sections were immunostained for E-cadherin (E-cad, magenta). Inset in A shows the anterior tongue region at a higher magnification. Dashed lines separate the tongue epithelium from the underlying mesenchyme. Scale bars: 50 μm. (B-F) Representative light microscopy images of E12 tongues (B,C) or E12+2-day tongue cultures (D-F) from *Cre^−^/Alk3^fx/fx^* (B), *Sox10-Cre/Alk3 cKO* (C) or wild type (D-F) mice. Tongues were immunostained for Shh (blue). Arrowheads and the arrow in B indicate Shh^high^ fungiform and circumvallate papilla placodes, respectively. In D-F, tongue cultures were treated with proteins from mesenchyme-conditioned medium (Mes-CM) of *Cre^−^/Alk3^fx/fx^* (Ctrl) or *Sox10-Cre/Alk3 cKO* (*cKO*), with or without 5 mM LiCl. The schematic diagrams illustrate how the experiment was set up. Scale bars: 200 µm.

To further confirm that the loss of taste papilla phenotype is indeed caused by the mesenchyme-specific deletion of *Alk3*, another *Cre* driver, *Sox10-Cre* was used (Yu et al., 2020; Matsuoka et al., 2005). *Sox10-Cre*-labeled cells were exclusively distributed in the tongue mesenchyme in serial sections of E12.0 *Sox10-Cre/nTnG* tongues (Fig. 6A). Like *Wnt1-Cre/Alk3 cKO*, a complete loss of taste papillae was observed in the *Sox10-Cre/Alk3 cKO* mice (Fig. 6C versus B). In addition, *Sox10-Cre/Alk3 cKO* also depicted a truncated tongue tip (Fig. 6C), and no significant changes of cell proliferation or apoptosis in the tongue epithelium and mesenchyme (Fig. S8). Furthermore, the protein extracted from *Sox10-Cre/Alk3 cKO* Mes-CM was potent enough to inhibit taste papilla development in WT tongue cultures (Fig. 6D-E). Such inhibition was reversed by the addition of LiCl (Fig. 6F).

### Mesenchymal *Alk3 cKO* alters the production of previously unappreciated secretory proteins

**Fig. 7.**
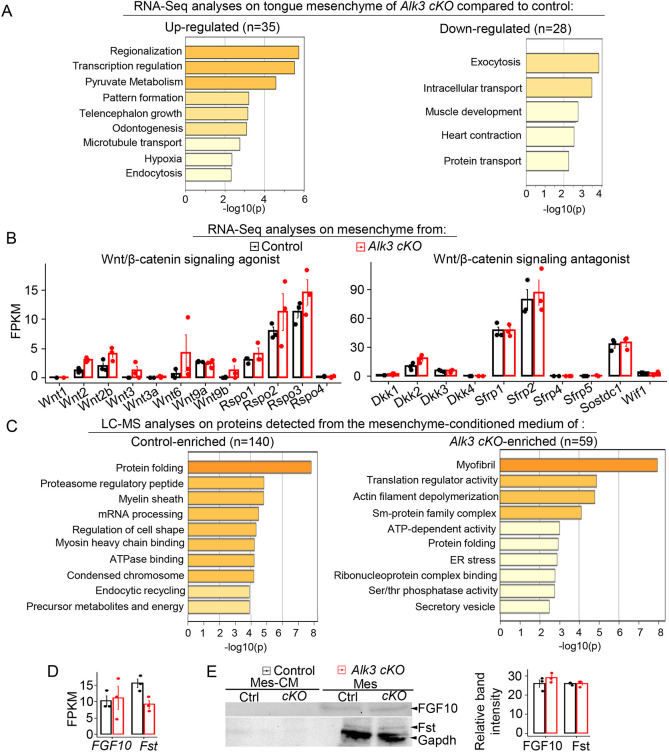
**Mesenchymal *Alk3 cKO* alters the production of proteins that have not previously been described.** (A) GO (Gene Ontology) enrichment and KEGG (Kyoto Encyclopedia of Genes and Genomes) pathway analyses to present the functional associations of RNA-sequencing-detected DEGs in the tongue mesenchyme of E12.0 *Wnt1-Cre/Alk3 cKO* (*Alk3 cKO*) compared with *Cre^−^/Alk3^fx/fx^* littermates. (B) Histograms (mean±s.d.; *n*=3) presenting the FPKM values of the known regulators of Wnt/β-catenin signaling in the E12.0 tongue mesenchyme. Control, *Cre^−^/Alk3^fx/fx^*; *Alk3 cKO*, *Wnt1-Cre/Alk3 cKO*. No statistically significant differences were found with (adjusted *P*-value) DESeq2 statistical analysis based on read counts. (C) GO enrichment and pathway analyses to show the functional association of differentially expressed proteins in the tongue mesenchyme-conditioned medium from *Wnt1-Cre/Alk3 cKO* (*Alk3 cKO*) and *Cre^−^/Alk3^fx/fx^* (control). These extracted proteins were detected with liquid chromatography-mass spectrometry (LC-MS). (D) A histogram (mean±s.d.; *n*=3) presenting the transcripts levels (FPKM values) of *Fgf10* and *Fst* (follistatin) in the tongue mesenchyme of *Cre^−^/Alk3^fx/fx^* (control) and *Wnt1-Cre/Alk3 cKO* (*Alk3 cKO*) mice. (E) Western blot bands of FGF10, Fst and Gapdh in the mesenchyme-conditioned medium (Mes-CM) or mesenchyme (Mes) tissue from E12.0 *Cre^−^/Alk3^fx/fx^* (Ctrl) and *Wnt1-Cre/Alk3 cKO* (*cKO*). The histogram on the right (mean±s.d.; *n*=3) presents the normalized band intensities of FGF10 and Fst relative to Gapdh in E12.0 *Cre^−^/Alk3^fx/fx^* (control) and *Wnt1-Cre/Alk3 cKO* (*Alk3 cKO*) tongue mesenchyme. No statistically significant differences were found in *Alk3 cKO* compared with the *Cre^−^/Alk3^fx/fx^* littermate controls using an unpaired Student's *t*-test.

To identify the tongue mesenchyme-produced proteins that regulate epithelial cell differentiation, we compared the transcript levels of protein-encoding genes in the tongue mesenchyme and proteomic profiles of Mes-CM between *Wnt1-Cre/Alk3 cKO* and control. Kyoto Encyclopedia of Genes and Genomes (KEGG) and Gene ontology (GO) analyses of RNA-sequencing data revealed that the 30 upregulated and 28 downregulated genes in E12.0 *Alk3 cKO* mesenchyme are associated with multiple biological processes. DEGs were enriched in protein trafficking, including intracellular transport and exocytosis, in addition to other development-related processes (Fig. 7A). The transcript level of Wnt/β-catenin signaling regulators, including agonists (Wnt ligands and R-spondins 1-4) and antagonist genes (*Dkk1*, *Dkk2*, *Dkk3*, *Dkk4*, *Sfrp1*, *Sfrp2*, *Sfrp3*, *Sfrp4*, *Sfrp5*, *Sostdc1* and *Wif1*) (MacDonald et al., 2009), were not altered in the E12.0 *Wnt1-Cre/Alk3 cKO* mesenchyme (Fig. 7B).

Out of the LC-MS-detected proteins in the Mes-CM, 199 proteins were differentially expressed (control or *Alk3 cKO* only). The proteomic profiles of Mes-CM were significantly altered by mesenchymal *Alk3 cKO*, among which 140 were enriched in control and 59 in *Wnt1-Cre/Alk3 cKO* group (Fig. 7C). GO analysis revealed that detected proteins in the *Wnt1-Cre/Alk3 cKO* group are mainly involved in post-transcriptional processes, including translational regulatory activity, post-translational modification (such as regulation of endoplasmic reticulum stress), protein folding and secretion, including secretory vesicles (Fig. 7C). Of note, none of the Wnt/β-catenin signaling agonists or antagonists was detected by the LC-MS. Moreover, the two mesenchymal secretory proteins (i.e. FGF10 and follistatin/Fst) that are known to regulate taste papilla development (Beites et al., 2009; Petersen et al., 2011; Prochazkova et al., 2017) appeared to be unaltered at both mRNA and proteins levels in the E12.0 *Wnt1-Cre/Alk3 cKO* mesenchyme compared with the littermate controls (Fig. 7D,E).

## DISCUSSION

**Fig. 8.**
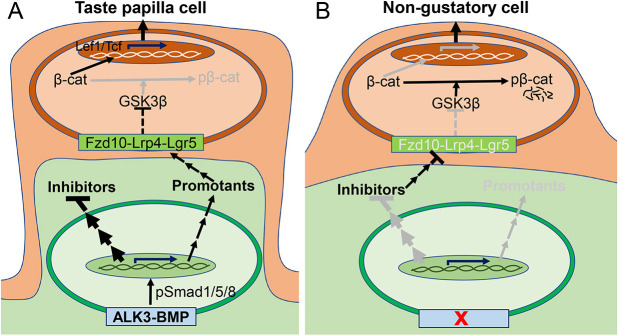
**The proposed model of how mesenchymal ALK3-BMP interacts with epithelial Wnt/β-catenin signaling for taste papilla cell differentiation.** (A,B) We propose that ALK3-BMP signaling in the tongue mesenchyme regulates the production of secretory proteins that promote or inhibit epithelial cell differentiation to taste papilla cells. Under normal conditions (A), mesenchymal ALK3-BMP signaling inhibits the secretion of inhibitors while enhancing the secretion of promoters that alter the Wnt/β-catenin components upstream of GSK3β and, thus, promote epithelial Wnt/β-catenin signaling. (B) Deletion of ALK3-BMP signaling leads to the enhanced production of inhibitors and, thus, to decreased Wnt/β-catenin activity and loss of taste papillae. Black and grey lines represent the active and inactive pathways, respectively; arrows and blocks represent activation and inhibition, respectively.

Our present study provides data for the first time demonstrating the requirement of ALK3-BMP signaling in the tongue mesenchyme for the proper activity of the Wnt/β-catenin pathway in the epithelium and for taste papilla cell differentiation during early embryonic development. We propose a working model describing how mesenchymal ALK3-BMP signaling interacts with the epithelial Wnt/β-catenin pathway to regulate taste papilla differentiation (Fig. 8). Under normal conditions, mesenchymal ALK3-BMP signaling facilitates the secretion of proteins that promote taste papilla cell differentiation and suppresses those inhibitory proteins, thus allowing the proper activity of epithelial Wnt/β-catenin signaling and taste papilla development. However, in the absence of mesenchymal ALK3-BMP signaling, the production of inhibitory secretory proteins is enhanced, which causes the deficiencies of epithelial Wnt/β-catenin signaling upstream to GSK3β, thereby resulting in the absence of taste papillae.

### Tongue mesenchyme determines epithelial cell fate at early stages of taste papilla development through the production of previously unreported secretory proteins

The importance of mesenchymal-epithelial interactions in the cell differentiation of epithelial appendages has been well documented for the development of many organs, including skin (Yamaguchi et al., 2004), mammary gland (Cunha and Hom, 1996), lung (Horowitz and Thannickal, 2006), kidney (Müller et al., 1997) and urogenital buds (Jerman et al., 2015). Unlike these other epithelial appendages, taste papillae are understudied with respect to the role of the underlying mesenchyme in their cell differentiation. Studies on molecular regulation of taste papilla cell differentiation have focused on the roles of signaling pathways within the epithelium, including multiple morphogens/growth factors (Castillo et al., 2014; Castillo-Azofeifa et al., 2017; Gaillard and Barlow, 2011; Gaillard et al., 2017, 2015; Iwatsuki et al., 2007; Liu et al., 2007, 2008; Thirumangalathu and Barlow, 2015; Petersen et al., 2011; Prochazkova et al., 2017; Zhou et al., 2006). It has been reported that the molecular programs in the epithelium determine the epithelial cell fate and the position of fungiform taste papillae at E13.5 (Kim et al., 2003), when taste papillae protrude from the tongue dorsum (Kaufman, 1992) and remain their stereotypic locations thereafter (Farbman and Mbiene, 1991; Paulson et al., 1985).

In the present study, we found that at the early stage (E12), when epithelial cells are rapidly differentiating, the molecular programs in the tongue epithelium are governed by the signals from the underlying mesenchyme. Specifically, knockout of a single gene encoding the type I BMP receptor *Alk3* in the neural crest (NC)-derived tongue mesenchyme leads to many differentially expressed genes (DEGs) in the tongue epithelium, which in turn leads to an absence of taste papillae. These data indicate the requirement of NC-derived tongue mesenchyme in taste papilla cell differentiation under the regulation of ALK3-BMP signaling pathway.

Regarding the factors that mediate mesenchymal-epithelial interactions, it has been reported that mesenchymal FGF10, a ligand in the FGF signaling pathway, and follistatin, a BMP antagonist, regulate the pattern and size of taste papillae in a region-specific manner (Petersen et al., 2011; Prochazkova et al., 2017; Beites et al., 2009). Our data reveal that mesenchyme-specific *Alk3 cKO* does not alter FGF10 and follistatin expression in the tongue mesenchyme at both transcript and protein levels. Instead, the production of many other proteins is changed, and proteins regulating taste papilla differentiation are within the range of 10-100 kDa. The results support the observations that many previously unreported proteins from the tongue mesenchyme signal to the overlying epithelium to regulate the cell differentiation.

Regarding the functions of these previously unreported mesenchyme-produced proteins, our data indicate two groups playing opposing roles: promoting or inhibiting taste papilla differentiation. Under normal conditions, tongue mesenchymal cells secrete 10-100 kDa proteins that promote taste papilla differentiation. In contrast, proteins (also 10-100 kDa) from *Alk3 cKO* tongue mesenchyme have profound inhibitory effects. Of note, such inhibition of taste papillae occurred in cultured wild-type tongues, in which normal mesenchyme was intact and present, indicating that the ‘inhibitory’ proteins from *Alk3 cKO* tongue mesenchyme are potent enough to overwrite the effects of promoters from wild-type mesenchyme. This indicates the necessity of ALK3-BMP signaling to serve as a ‘brake’, inhibiting the production of these ‘inhibitory’ proteins for taste papilla formation. Furthermore, constitutive activation of ALK3 does not enhance taste papilla cell differentiation, supporting the observation that normal ALK3-BMP activity is robust and highly active so that the inhibitory proteins are fully inhibited; thus, having *caAlk3* does not enhance the inhibition further. Moreover, the *ex vivo* administration of 10-100 kDa proteins from wild-type tongue mesenchyme may represent an addition of a high dose of isolated protein fraction that contain active ‘promoters’. *In vivo* constitutive activation of *Alk3* likely does not enhance the protein production to this level to depict the *ex vivo* phenotype in promoting taste papilla formation. Together, our data support the idea that ALK3-BMP signaling in the tongue mesenchyme promotes taste papilla cell differentiation through governing the production of secretory proteins, i.e. suppressing the inhibitors while enhancing the promoters.

Regarding how ALK3-BMP signaling regulates the production of secretory proteins, it is important to note that *Alk3 cKO*-induced alterations of transcripts in the tongue mesenchyme and of proteins in the *Alk3 cKO* Mes-CM did not overlap. Although we are aware that the concerns regarding the need for technical optimization cannot be excluded, it is plausible to speculate that ALK3-BMP signaling in the mesenchyme affects the protein production at post-transcriptional levels (e.g. translational regulation, post-translational modifications and secretions).

The homogeneous epithelial cells at E11-E11.5 undergo rapid differentiation to form the taste papilla placodes and non-gustatory cells between papillae (Barlow, 2015; Kramer et al., 2019). At E11.0-E11.5, all epithelial cells in the lateral tongue swellings are Shh^high^Krt8^+^. Within a short time period (∼12 h), the cells either remain as Shh^high^Krt8^+^ clusters and adopt a gustatory/taste papilla cell fate, or become Shh^low^Krt8^−^ non-gustatory cells (Kramer et al., 2019). Our findings clearly indicate that the mesenchyme promotes the gustatory cell fate of the tongue epithelium via ALK3-BMP signaling, while inhibiting its non-gustatory cell fate. Furthermore, in the cultures of E12 wild-type tongues in which the epithelial cells have already acquired their gustatory or non-gustatory cell fate, *Alk3 cKO* tongue mesenchyme-produced proteins are potent enough to transform the epithelial cell fate.

### Mesenchymal BMP signaling regulates taste papilla differentiation in a receptor-specific manner

The importance of BMP signaling in regulating taste papilla cell differentiation has been documented (Ishan et al., 2020; Beites et al., 2009; Zhou et al., 2006). However, the receptors that mediate it and the signals involved from specific tissue compartments are not clear. In the present study, we detected the four types of type I BMP receptors at different transcripts levels (*Alk3*≫*Alk2*>*Alk6*≈*Alk1*), with the *Alk3* being expressed far more highly than the other three types. Further phenotypic analyses using mesenchyme-specific knockout of *Alk3* and *Alk2* demonstrated that the BMP signaling mediated by ALK3, not ALK2, is essential for taste papilla cell differentiation, revealing receptor-specific roles of BMP signaling in the tongue mesenchyme in regulating tongue epithelial cell differentiation.

Regarding the intracellular signaling pathway, our data reveal that it is most likely through p-Smad1/5/8 that ALK3-BMP signaling regulates taste papilla differentiation. First, p-Smad1/5/8 signals in the tongue mesenchyme and epithelium are robustly detected as early as at E12.0, earlier than the reported starting point (E14.5) in previous studies (Kawasaki et al., 2012). Second, the proteins extracted from the conditioned medium of mesenchymal cells treated with p-Smad1/5/8 inhibitor dorsomorphin mimics the taste papilla loss caused by *Alk3 cKO* tongue mesenchyme in cultures. Furthermore, the previous reports (Liu et al., 2018; Yumoto et al., 2013) showed that taste papillae form after the loss of function of non-canonical (pSmad1/5/8-independent) BMP signaling. Together, these data reveal that ALK3-BMP signaling regulates taste papilla differentiation via pSmad1/5/8. Of note, Smad1, Smad5 and Smad8 are expressed at different levels, with a lower FPKM value for Smad8 than for Smad1 and Smad5. The immunosignals of p-Smad1/5/8 in the tongue mesenchymal cells cannot be distinguished from one another. Studies using the specific knockout of each factor (Smad1, Smad5 or Smad8) for a comparison will be beneficial to drawing a conclusion.

The effects of BMPs on taste papilla formation are stage specific, i.e. promoting taste papilla cell differentiation in rat tongue cultures starting at E13 (≈mouse E11.5) while inhibiting at E14 (≈mouse E12.5) (Zhou et al., 2006). The absence of taste papillae in mesenchymal *Alk3 cKO* mice is consistent with (1) the promoting effects of BMP ligands (BMP2, BMP4 and BMP7) on taste papilla development at the early embryonic stage and (2) the inhibitory roles of BMP antagonist follistatin (Beites et al., 2009). It is intriguing that another BMP antagonist Noggin promotes taste papilla cell differentiation in both E13 and E14 rat tongue cultures (Zhou et al., 2006). It is possible that Noggin exerts its roles independently of conventional BMP signaling (Bernatik et al., 2017), or that, in the 2-day tongue cultures, the promoting effect of Noggin that occurs later overwrites its effects at the initial earlier stage. A delicately designed experiment is needed to test which of the possibilities is true. As for the inhibitory effect of BMP ligands on taste papilla formation at the later stage (rat E14), further studies are needed for a clear understanding of the mediating signaling cascades.

It has been reported that *Alk3* deletion may cause the death of NC cells immediately after normal cell migration to their destination (e.g. the dorsal aorta) (Morikawa et al., 2009). To address whether deficient taste papilla cell differentiation in *Alk3 cKO* is caused by the lack of tongue mesenchyme, we analyzed the phenotypic changes at early embryonic stages. At E10.5, when the NC-derived cells massively populate the primordia of tongue organ (Han et al., 2012; Ishan et al., 2020), the tongue primordia (i.e. branchial arches I-IV) developed in *Alk3 cKO* similarly to the littermate control. Furthermore, both *in vivo* and *ex vivo* analyses demonstrate that tongue mesenchymal cells are highly proliferating and not apoptotic in *Alk3 cKO*. Our data support the observation that the absence of taste papilla cells in the epithelium is not due to the missing mesenchyme, but rather is caused by the products of the mesenchyme that are the secretory proteins, as discussed above.

After NC-derived cells populate the tongue mesenchyme, non-NC-derived cells, such as myoprogenitors start to migrate and differentiate into the tongue muscles (Han et al., 2012; Millington et al., 2017; Parada et al., 2012). The myoprogenitors emerge at the core of the mesenchyme and eventually spread throughout the body of the tongue leaving the layer adjacent to the epithelium as a dense population of NC-derived mesenchymal cells (Liu et al., 2012a; Thirumangalathu et al., 2009). Thus, *Alk3 cKO* in the NC-derived tongue mesenchyme caused the absence of *Alk3* transcripts mainly in the mesenchyme closer to the epithelium, as shown by the *in situ* hybridization signals. The broad expression of *Alk3* in non-NC-derived myoprogenitors could be the reason that a comparable level of *Alk3* transcripts are detected in NC-derived mesenchyme-specific *Alk3 cKO* compared with controls. The papilla loss in *Alk3 cKO* indicates that ALK3-BMP signaling in the NC-derived mesenchymal cells, but not in the non-NC-derived myoprogenitors, is required for taste papilla differentiation. We speculate that the mesenchyme immediately beneath the epithelium plays a crucial role in regulating epithelial cell differentiation.

### Wnt/β-catenin signaling in tongue epithelium is a downstream target of mesenchymal ALK3-BMP signaling in regulating taste papilla differentiation

The Wnt/β-catenin pathway is essential for taste papilla development (Liu et al., 2007; Iwatsuki et al., 2007; Zhu et al., 2014; Thirumangalathu and Barlow, 2015). Without Wnt ligands, cytoplasmic β-catenin is constitutively degraded by the destruction complex composed of axin, adenomatosis polyposis coli (APC), protein phosphatase 2A (PP2A), casein kinase 1α (CK1α) and glycogen synthase kinase 3β (GSK3β) (Komiya and Habas, 2008). Phosphorylation of β-catenin by CK1α and GSK3β leads to ubiquitin-mediated proteolytic destruction, thus making it unavailable for nuclear translocation (Komiya and Habas, 2008). Binding of Wnt ligands to the receptor complex composed of the Frizzled (Fzd) and the lipoprotein receptor-related protein (LRP)5/6 promotes Axin translocation to the cytoplasmic tail of LRP5/6 and activates disheveled (Dvl), and, in turn, inhibits GSK3β. Therefore, β-catenin proteins may be stabilized and translocated to the nucleus to serve as co-transcription factors for Lef1/Tcf (Komiya and Habas, 2008) and/or Prox1 (Liu et al., 2015) in regulating target gene expression.

In the present study, our data indicate that the downregulation of Wnt/β-catenin signaling is the main cause of taste papilla loss in the mesenchymal *Alk3 cKO*. The expression of Wnt/β-catenin signaling components in the epithelium is altered, including the downregulation of three encoding genes of key positive regulators (*Fzd10*, *Lgr5* and *Prox1*) and the upregulation of the inhibitor *Sostdc1* (sclerostin domain-containing 1) (Lintern et al., 2009). The reduction of Wnt/β-catenin activity is confirmed by the lack of nuclear accumulation of β-catenin in the tongue epithelium of *Alk3 cKO*. Addition of GSK3β inhibitor LiCl (Iwatsuki et al., 2007) to activate Wnt/β-catenin pathway rescues taste papillae in *Alk3 cKO* tongue cultures. We did not detect altered transcripts and protein levels of Wnt/β-catenin agonists and antagonists in *Alk3 cKO* compared with control. Moreover, addition of Wnt3a to the culture medium did not rescue taste papilla formation in *Alk3 cKO* while effectively promoting taste papilla formation in controls. Together, our data indicate a defective epithelial Wnt/β-catenin pathway upstream of GSK3β in *Alk3 cKO*, which may be due to the lack of receptors Lgr5 and Fzd10, and to the increase in the inhibitor Sostdc1.

### Tongue and mandibular outgrowth are regulated by separate molecular programs

During the tongue organogenesis, the three lingual swellings (two lateral and one posterior – tuberculum impar) emerge on the floor of the mandible (Cobourne et al., 2019). As the tongue and mandible are structurally connected, close coordination is essential (Hutchinson et al., 2014; Parada and Chai, 2015). While the lingual swellings merge to form the spatulate tongue around E12, simultaneous development takes place in the mandibular primordium, extending downwards and elongating (Ramaesh and Bard, 2003; Cobourne et al., 2019). This concurrent growth is essential for the development of both tongue and mandible. Previous research has shown that tongue and mandibular outgrowth are regulated by multiple but different molecular signaling pathways.

For the tongue outgrowth, Wnt, TGFβ, Shh, BMP and NF2/Hippo signaling are known to play important roles. Genetic manipulations targeting these pathways have shown significant effects on tongue development. For example, mesenchymal-specific deletion of the *TGFBR2* gene in TGFβ signaling and loss of KiF3a and intraflagellar proteins in Shh signaling lead to microglossia (Hosokawa et al., 2010; Iwata et al., 2013; Parada et al., 2012) and aglossia (Millington et al., 2017), respectively. Moreover, the absence of Hand2, a basic helix-loop-helix transcription factor, in neural crest cell lineages leads to aglossia through regulating Dlx5 and Dlx6 expression (Barron et al., 2011). Our previous research has shown that *Wnt1-Cre*-mediated constitutive activation of *Alk2* leads to microglossia (Ishan et al., 2020), whereas the conditional knockout of *Nf2* results in macroglossia (Ishan et al., 2021) during the early stages of embryonic development. Moreover, proper levels of Wnt5a in the mesenchyme are essential for tongue outgrowth (Liu et al., 2012b), as the *Wnt5a* knockout mice presented with a shorter tongue with ankyloglossia. These findings indicate the complexity of the precise molecular regulation needed to control tongue outgrowth.

As for the outgrowth of the mandible/lower jaw, multiple signaling pathways are involved, including BMPs (Matsui and Klingensmith, 2014; Wang et al., 1999; Stottmann et al., 2001), Wnt/β-catenin (Li et al., 2017) and EGF (Miettinen et al., 1999). For example, BMP antagonists Chordin and Noggin are essential in suppressing BMP activity while upregulating FGF8 for the cell survival during mandibular outgrowth (Stottmann et al., 2001). Moreover, ISLET1 in the mandibular epithelium is required for the activation of epithelial β-catenin and Shh signals that subsequently impact on the mesenchymal cell survival and outgrowth of the mandible (Li et al., 2017). In addition, newborn EGFR null mutant mice have an underdeveloped lower jaw (Miettinen et al., 1999).

In the present study, *Alk3 cKO* in the NC-derived mesenchyme caused truncation of mandible and tongue tip. However, in culture conditions when the tongue is free from the constraints imposed by the mandible, the tongue elongates and outgrows well. Our results indicate that the truncation of the tongue tip is caused by the truncated mandible, not by a developmental defect alone. Taken together, the previously published and our current data infer that the tongue and mandibular outgrowth are regulated by separate signaling pathways. The importance of BMP signaling in mandibular development is supported by the data from another study that demonstrates the requirement of Noggin for the proper outgrowth of the mandibular bud (Matsui and Klingensmith, 2014). Our results in the current study demonstrate that ALK3-BMP signaling is crucial for the development of the mandible.

In summary, our findings propose a previously unreported concept in the field of taste biology: during early embryonic development, taste papilla cell differentiation requires mesenchymal ALK3-BMP signaling to suppress the production of inhibitory secretory proteins, thus allowing proper activation of epithelial Wnt/β-catenin signaling and taste papilla development. Additionally, we have found that ALK3-BMP in the mesenchyme also plays a crucial role in mandible development. It will be important to define the roles of mesenchymal stromal cells and cell products in regulating taste cell renewal of mature taste buds. If this new concept is found to be true in adults, identification of these regulatory proteins may be beneficial to developing novel therapeutic treatments for taste disorders caused by deficiencies in taste cell differentiation.

## MATERIALS AND METHODS

### Animal use and tissue collection

The use of animals was approved by the Institutional Animal Care and Use Committee at the University of Georgia. The studies were performed in compliance with the National Institutes of Health Guidelines for the care and use of animals in research. The animals were maintained in the animal facilities in the Department of Animal and Dairy Science at the University of Georgia.

Wild type (C57BL/6J, stock #000664) and nuclear tdTomato nuclear EGFP (nTnG) double reporter [B6; 129S6-Gt-GT(ROSA) 26Sor^tm1(CAG-tdTomato*, EGFP*)Ees^/J, stock 023035] mice were purchased from the Jackson Laboratory. *Alk2 floxed* (*Alk2^fx/fx^*) (Kaartinen and Nagy, 2001) and *Alk3 floxed* (*Alk3^fx/fx^*) (Mishina et al., 2002) mice, and mice carrying a constitutively active form of the *Alk3* transgene (*CAG-Z-EGFP-caAlk3*; hereafter *caAlk3*) (Komatsu et al., 2013) were provided by Dr Yuji Mishina (University of Michigan, USA). Heterozygous *Wnt1-Cre* [B6.Cg-^Tg(Wnt1-Cre)11Rth^ Tg(Wnt1-GAL4) 11Rth/J, Jackson Laboratory, 003829] or *Sox10-Cre* [B6; CBA-Tg (Sox10-Cre) 1Wdr/J, 025807] mice were bred with homozygous *Alk2^fx/fx^* or *Alk3^fx/fx^* mice to generate *Wnt1-Cre/Alk2^fx/+^*, *Wnt1-Cre/Alk3^fx/+^* and *Sox10-Cre/Alk3^fx/+^* mice that were backcrossed with *Alk2^fx/fx^* and *Alk3^fx/fx^* mice to generate conditional knockout (cKO) embryos (*Wnt1-Cre/Alk2 cKO*, *Wnt1-Cre/Alk3 cKO* and *Sox10-Cre/Alk3 cKO*). To generate *Wnt1-Cre/caAlk3* mice, heterozygous *Wnt1-Cre* mice were bred with homozygous *caAlk3* mice. *Cre*-negative (*Cre^−^*) littermates (*Cre^−^/Alk2^fx/fx^*, *Cre^−^/Alk3^fx/fx^* or *Cre^−^/caAlk3*) were used as controls.

Noon of the day on which the dam was positive for a vaginal plug was designated as embryonic day (E) 0.5. Timed pregnant mice were euthanized with CO_2_ followed by cervical dislocation. Embryos (E10.5-E14.5) were dissected from the uterus under a dissection microscope. The stages of embryos were confirmed by the number of somite pairs and the development of multiple organs. To label cells with 5-bromo-2′-deoxyuridine (BrdU), a dose of 100 mg/kg body weight of BrdU (10280879001, Millipore Sigma) was intraperitoneally injected into the dam 2 h before harvesting the embryos.

The following primers were used for genotyping: 5′-CCCCCATTGAAGGTTTAGAGAGAC-3′ and 5′-CTAAGAGCCATGACAGAGGTTG-3′ for *Alk2 floxed* (160 bp) and wild-type (250 bp) fragments; 5′-GCAGCTGCTGCTGCAGCCTCC-3′ and 5′-TGGCTACAATTTGTCTCATGC-3′ for *Alk3 floxed* (230 bp) and wild-type (150 bp) fragments; 5′-GTGCTGGTTATTGTGCTGTCTC-3′ and 5′-GACGACAGTATCGGCCTCAGGAA-3′ for *caAlk3* gene product (580 bp; Yang and Mishina, 2019); and 5′-ATTGCTGTCACTTGGTCGTGGC-3′ and 5′-GGAAAATGCTTCTGTCCGTTTGC-3′ for the *Cre* gene product (200 bp).

### Immunohistochemistry on whole-mount organs

Embryonic (E10.5-E14.5) tongues and E12.0+2-day tongue organ cultures were processed for immunohistochemistry as previously described (Liu et al., 2004). Briefly, tongue organs or cultures were fixed in 4% paraformaldehyde (PFA) at 4°C for 2 h and then washed in 0.1 M phosphate-buffered saline (PBS). Blocking of endogenous hydrogen peroxidase was performed using 6% H_2_O_2_ in methanol followed by an antigen retrieval process, i.e. heating at 95°C for 5 min in antigen retrieval solution (CTS045, R&D Systems). After blocking non-specific binding in 2% non-fat milk in 0.1 M PBS containing 0.1% Triton X-100 (PBS/MT; X-100, Sigma Aldrich), the organs were incubated in goat anti-Shh primary antibody (Table S6) in 10% normal donkey serum (NDS; D9663, Sigma Aldrich) in PBS/MT at 4°C for 48 h. After thorough washing in PBS/MT on ice (1 h×5), the organs were incubated in biotin-conjugated secondary antibody (1:500; BA-5000, Vector Laboratories) in 1% NDS in PBS/MT overnight at 4°C and subjected to peroxidase-conjugated streptavidin treatment in blocking solution (1:500; PK6200, Vector Laboratories) overnight at 4°C after rinsing in PBS/MT on ice (1 h×5). Following rinses in PBS/MT (1 h×5) and PBT (0.1 M PBS, 0.1% Triton X-100 and 0.2% bovine serum albumin; 1 h×2) on ice, tongue organs were processed for DAB (SK4100, Vector Laboratories) pre-incubation (without H_2_O_2_) and reaction (with H_2_O_2_). The immunostained tongues were rinsed in 0.1 M PBS and photographed using a SZX16 Olympus Stereomicroscope.

### Immunohistochemistry on cells and sections

E11.5 mesenchymal cells or E12.0-E14.5 tongue tissues were fixed in 4% PFA in 0.1 M PBS at 4°C for 15 min (cells) or 2 h (tongues). PFA-fixed tongue tissues were cryoprotected in 30% sucrose in 0.1 M PBS at 4°C for at least 24 h, embedded in OCT compound (23730571, Fisher Scientific) and rapidly frozen. Cryostat sections were cut at 10 μm for immunohistochemistry. Tongue sections were air-dried at room temperature for 1 h, then rehydrated in 0.1 M PBS (5-10 min×3).

Blocking of nonspecific binding in cells or tongue sections was carried out by incubation with 10% NDS in 0.1 M PBS containing 0.3% Triton X-100 at room temperature for 30 min. The cells or sections were then incubated with primary antibodies (Table S6) in carrier solution (1% normal donkey serum, 0.3% Triton X-100 in 0.1 M PBS) at 4°C for overnight. Cells and sections treated without a primary antibody were used as negative controls. After rinsing in 0.1 M PBS (three times for 5-10 min) at room temperature, cells or sections were incubated with Alexa Fluor 488- or 647-conjugated secondary antibody (1:500; Invitrogen) in carrier solution at room temperature for 1 h, rinsed with 0.1 M PBS (three times for 5-10 min) at room temperature and then counterstained with DAPI (200 ng/ml in PBS; D1306, Life Technologies) at room temperature for 10 min. After thorough rinsing in 0.1 M PBS, cells or sections were air-dried and mounted with Prolong Diamond antifade mounting medium (P36970, Fisher Scientific). Immunostained sections and cells were examined under a fluorescent light microscope (EVOS FL, Life Technologies). A laser scanning confocal microscope (Zeiss LSM 710, Biomedical Microscopy Core at the University of Georgia) was used to take single-plane images.

### Immunohistochemistry on epithelial sheets

The tongue epithelium was separated from the mesenchyme in postnatal day 21 (P21) *Wnt1-Cre/caAlk3* and *Cre^−^* littermate control mice, as previously described (Liu et al., 2008; Venkatesan et al., 2016). Separated epithelial sheets were immunostained for pan taste cell marker Krt8 (Table S6), as previously described (Liu et al., 2008; Ishan et al., 2020; Venkatesan et al., 2016), and photographed using an SZX16 Olympus Stereomicroscope.

### *In situ* hybridization

Tongues were dissected from E12.0 *Wnt1-Cre/Alk3 cKO* and *Cre^−^* littermate control embryos in 0.1 M PBS and fixed with 4% PFA at 4°C for 24 h. PFA-fixed tissues were processed for cryostat sectioning at 15 μm. Conventional *in situ* hybridization for *Alk3* was performed as previously described (Lauter et al., 2011) using digoxigenin-labeled riboprobes. Plasmids carrying the *Alk3* probe were provided by Dr Yuji Mishina (University of Michigan, USA). Digoxigenin-labeled antisense *Alk3* RNA probe was prepared by linearization with EcoR1 (New England Biolabs) and transcription with T7 RNA polymerase (Promega).

For RNAscope *in situ* hybridization, RNAscope Intro Pack 2.5 HD Reagent Kit Brown (322300, Advanced Cell Diagnostics) was used on E12.0 wild-type tongue sections. *Alk2* (312411) and *Alk3* (312421) probes were purchased from Advanced Cell Diagnostics and *in situ* hybridization was performed by following the manufacturer's instructions. Cell nuclear counterstaining was performed with 50% Hematoxylin.

### Scanning electron microscopy (SEM)

E10.5-E11.5 *Cre^−^* littermate control and *Wnt1-Cre/Alk3 cKO* branchial arches (BAs) or tongues were fixed in 2.5% glutaraldehyde (#75520; Electron Microscopy Science, Hatfield, PA) and 4% PFA in 0.1 M PBS (pH 7.3) at 4°C for 24 h. After rinsing in 0.1 M PBS at room temperature (three times for 10 min), tissues were post-fixed in a sequence of aqueous 1% O_S_O_4_ (19150, Electron Microscopy Science) in 0.1 M PBS, 1% tannic acid (16201, Sigma Aldrich) in milliQ H_2_O and 1% O_S_O_4_ in milliQ H_2_O on ice for 1 h each. Tissues were then dehydrated in an ascending series of ethanol (35, 50, 70, 90 and 100%) and hexamethyldisilazane (HMDS, 440191; Sigma Aldrich) at room temperature (three times for 1 h). After a slow air dry in a fume hood, tissues were mounted on specimen stubs and sputter-coated with gold/palladium (Leica Gold/Carbon coater; Georgia Electron Microscope Core Facility, University of Georgia). Tissues were then imaged using a scanning electron microscope (FEI Teneo FE-SEM; Georgia Electron Microscope Core Facility, University of Georgia).

### Collection of conditioned media from mesenchymal cell cultures

The E11.5 tongue swellings were dissected from the mandible and the mesenchyme was separated from the epithelium, as previously described (Liu et al., 2008). At this stage, the mesenchyme is primarily populated by NC-derived cells (Han et al., 2012) and very few non-NC-derived myoprogenitors are present in the tongue mesenchyme (Han et al., 2012). Separated mesenchyme was then transferred to a culture dish, cut into small pieces and cultured in a humidified CO_2_ incubator at 37°C in a serum-free medium, i.e. a 1:1 mixture of Dulbecco's modified Eagle's medium and Ham's nutrient F12 (11320033, DMEM/F12, Gibco) containing 50 μg/ml gentamicin sulfate (15750060, Gibco). After 1 day in culture, the medium was replaced with fresh medium and continued to culture for 2 days. Culture medium was collected as conditioned medium. To inhibit the p-Smad1/5/8 activity, dorsomorphin (3093, R&D Systems) was added at 30 µg/ml to the cultures of mesenchyme cells from wild-type mouse tongue.

### Extraction of proteins and protein fractions from conditioned medium

Proteins were extracted from the conditioned medium using the protein precipitation kit (2100, Millipore Sigma) following the manufacturer's specifications. To isolate proteins at different molecular weights, mesenchyme-conditioned media were filtered through 100 kDa followed by 10 kDa Amicon filters (UFC910024 and UCF910008, Millipore Sigma) by centrifugation at 4000 ***g*** for 10 min to obtain >100 kDa and 10-100 kDa proteins as described previously (Whittaker et al., 2020). Proteins (<10 kDa) from the medium leaked through 10 kDa filters were extracted using the protein precipitation kit (2100, Millipore Sigma). The supernatant was used as the residual solution.

### Liquid chromatography-mass spectrometry analysis of proteins in mesenchyme-conditioned medium

Proteins isolated from the *Wnt1-Cre/Alk3 cKO* and *Cre^−^* littermate control conditioned medium were reduced with 5 mM of Tris (2-carboxyethyl) phosphine hydrochloride, alkylated with 13.75 mM of iodoacetamide and digested with trypsin/Lys-C mix (V5071, Promega). The resulting peptides were cleaned up with Acclaim PepMap 100 C18 spin columns (SEM SS18V, The Nest Group) dried down, and reconstituted in 0.1% formic acid. The reconstituted peptides were separated on an Acclaim PepMap 100 C18 column and eluted into the nano-electrospray ion source of an Orbitrap Eclipse Tribrid mass spectrometer (Thermo Fisher Scientific) at a flow rate of 200 nl/min. The elution gradient consists of 1-40% acetonitrile in 0.1% formic acid over 220 min followed by 10 min of 80% acetonitrile in 0.1% formic acid. The spray voltage was set to 2.2 kV and the temperature of the heated capillary was set to 275°C. The full mass spectrometry scans were acquired from m/z 300 to 2000 at 60 k resolution in the orbitrap, and the MS2 scans for the most intense precursors were fragmented via collision-induced dissociation (CID) and collected in the ion trap.

The raw spectra were searched against a mouse protein database (UniProt) by SEQUEST using Proteome Discoverer (v2.5, Fisher Scientific). The mass tolerance was set as 20 ppm for precursors and 0.5 Da for fragments. The peptide-spectrum matches (PSMs) resulting from the database search were identified, quantified and filtered to a 10% peptide false discovery rate (FDR) then clustered into a final protein-level FDR of 1%. The NSAF (Normalized spectral abundance factor) values were calculated for each protein and used to quantify their relative abundance and fold change across samples.

Gene Ontology (GO) enrichment and pathway analysis (http://www.geneontology.org/GO.database.shtml) was used to analyze the functional associations of the identified differentially expressed proteins (control only or *Alk3 cKO* only with ≥100 NSAF) from the mass spectrometry analyses.

### Tongue organ cultures

E12.0+2-day tongue organ cultures were prepared as previously described (Mbiene et al., 1997; Mistretta et al., 2003; Liu et al., 2004, 2008, 2012b; Zhou et al., 2006). To test the impact of tongue mesenchyme on epithelial cell differentiation and taste papilla formation, whole tongue organs were cultured to preserve the integrity of rapidly differentiating epithelial cells at E12. The mesenchymal tissue or mesenchyme-conditioned medium of E11.5-12.0 *Cre^−^* littermate control or *Wnt1-Cre/Alk3 cKO* tongues was added to E12.0 wild-type tongue cultures. Proteins from conditioned medium (>100 kDa, 10-100 kDa or <10 kDa at a concentration of 200 µg/ml) or residual solution were added to the standard culture medium. The *ex vivo* administration of proteins (>100 kDa, 10-100 kDa or <10 kDa) represents an addition of a high dose of isolated protein fraction.

To activate Wnt/β-catenin signaling, 5 mM LiCl (Clément-Lacroix et al., 2005) or 20% Wnt3a conditioned medium (J2-001, MBL international) was added to the culture medium. To digest proteins extracted from control and *Alk3 cKO* mesenchyme-conditioned medium, an equal amount of proteinase K (3115879001, Sigma Aldrich) (i.e. 200 µg/ml proteinase K for 200 µg/ml of proteins) was added and incubated at 37°C for 6 h. After the digestion, proteinase K was inactivated by heating at 95°C for 10 min, before adding the digested protein products into the culture medium. After 2 days, cultures were collected and processed for analyzing taste papilla formation and epithelial cell differentiation using Shh immunosignals in the tongue cultures.

### RNA sequencing and quantitative reverse transcriptase-polymerase chain reaction

E12.0 tongues were collected. At this stage, the taste papilla loss in *Alk3 cKO* is striking, the tongue mesenchyme is largely populated by *Wnt1-Cre*-labeled NC-derived cells (Han et al., 2012) and only a very small population of non-NC-derived myoprogenitors are present (Han et al., 2012). Separated mesenchymal and epithelial tissues from E12.0 *Cre^−^* littermate control and *Wnt1-Cre/Alk3 cKO* tongues were immersed in Trizol solution (15596018, Life Technologies) for RNA extraction using the RNeasy Plus kit (74136, Qiagen). For each experimental condition, a total of nine mesenchymal and epithelial tissues (pooled three tissues×three replicates) were used. RNA concentrations were measured using Nanodrop 8000 spectrophotometer (ThermoFisher Scientific).

RNA sequencing was performed in Georgia Genomics and Bioinformatics Center at the University of Georgia using the NextSeq 500 system (Illumina) following the procedures described previously (Ishan et al., 2021). Raw data were mapped to mouse reference genome GRCm38 (mm10) using STAR (Dobin et al., 2013). Transcripts were analyzed and reported as FPKM (fragments per kilobase per million mapped reads) by StringTie (Pertea et al., 2015). Differentially expressed genes (DEGs) were detected using the R package DESeq2 (Love et al., 2014). GO and Kyoto Encyclopedia of Genes and Genomes (KEGG) pathway analyses were used to investigate the functional associations of the DEGs. R package ggplot2 was used to illustrate data as histograms.

For qRT-PCR analyses, complementary DNA (cDNA) was synthesized from the extracted RNA using SuperScript First-Strand Synthesis System (11902018, ThermoFisher Scientific). The expression of the *Alk3* gene was detected using 5′-GACCAGAAGAAGCCAGAAAATGGA-3′ and 5′-TGTCCTGAGCAATAGCACTTTAAGAA-3′ primers (Yang and Mishina, 2019). Changes in gene expression levels in *Wnt1-Cre/Alk3 cKO* and *Cre^−^* littermate control tongue epithelium and mesenchyme are presented as mean±s.d.; *n*=3) of 2^−ΔCT^ values.

### Western blot

To detect the levels of proteins extracted from the E12.0 tongue mesenchyme and E11.5 mesenchyme-conditioned medium of *Wnt1-Cre/Alk3 cKO* and *Cre^−^* littermate control mice, SDS-PAGE and western blot were conducted as described previously (Ishan et al., 2021). Proteins from the tongue mesenchyme were extracted using radioimmunoprecipitation assay (RIPA) buffer [1% NP-40, 150 mmol/l NaCl, 50 mmol/l Tris-HCI, 0.5% sodium deoxycholate, 0.1% SDS and 1 mmol/l EDTA (pH 7.4)].

### Quantification and statistical analyses

To quantify the number of Ki67^+^, BrdU^+^, p-H3^+^, c-Cas3^+^ and p-Smad1/5/8^+^ cells per unit area (mm^2^) in tongue sections of E12.0 *Wnt1-Cre/Alk3 cKO* and *Cre^−^* littermate control mice (*n*=3 each group), serial sections were immunostained for Ki67, BrdU, p-H3, c-Cas3 and p-Smad1/5/8, and thoroughly analyzed under a fluorescent light microscope (EVOS FL, Life Technologies). Single-plane laser scanning confocal photomicrographs were taken from every other section using a laser scanning confocal microscope (Zeiss LSM 710, Biomedical Microscopy Core at the University of Georgia). Labeled cells were quantified on the sections of the anterior tongue region. For quantification of the number of fungiform papillae in the E12+2-day wild-type tongue cultures, bright-field images of anti-Shh immunostained tongues were used. A round patch of Shh^+^ epithelial thickening was counted as a fungiform papilla. To quantify the vimentin^+^ and Ki67^+^ cells, single-plane laser scanning confocal photomicrographs were taken from the vimentin and Ki67 immunostained E11.5+3-day cultures of mesenchymal cells from *Wnt1-Cre/Alk3 cKO* and *Cre^−^* littermate control tongues. Numbers of vimentin^+^ and Ki67^+^ cells were counted in relation to the total number of DAPI^+^ cells and presented as a percentage of vimentin^+^ and Ki67^+^ cells.

Data are presented as mean±s.d. (*n*=3). Two-way analyses of variance (ANOVA) followed by Fisher's LSD analyses were used to compare: (1) the numbers of fungiform papillae in E12+2-day wild-type tongue cultures; (2) Ki67^+^, BrdU^+^, p-H3^+^, c-Cas3^+^ and p-Smad1/5/8^+^ cells per unit area (mm^2^) in tongue epithelium and mesenchyme from *Wnt1-Cre/Alk3 cKO* and *Cre^−^* littermate mice; and (3) the percentage of vimentin^+^ and Ki67^+^ cells detected from cell cultures of *Wnt1-Cre/Alk3 cKO* and *Cre^−^* littermate tongue mesenchyme. An unpaired Student's *t*-test was used to evaluate the statistical significance of the differences between western blot band intensities. *P*<0.05 was considered to be statistically significant.

## Supplementary Material

10.1242/develop.201838_sup1 Supplementary information
