# Supplementary material for: Taste papilla cell differentiation requires the regulation of secretory protein production by ALK3-BMP signaling in the tongue mesenchyme
Source: Development. 2023 Sep 25;150(18):dev201838. doi: 10.1242/dev.201838 (PMC10560570; doi:10.1242/dev.201838)
Supplement: Supplementary information [file develop-150-201838-s1.pdf]

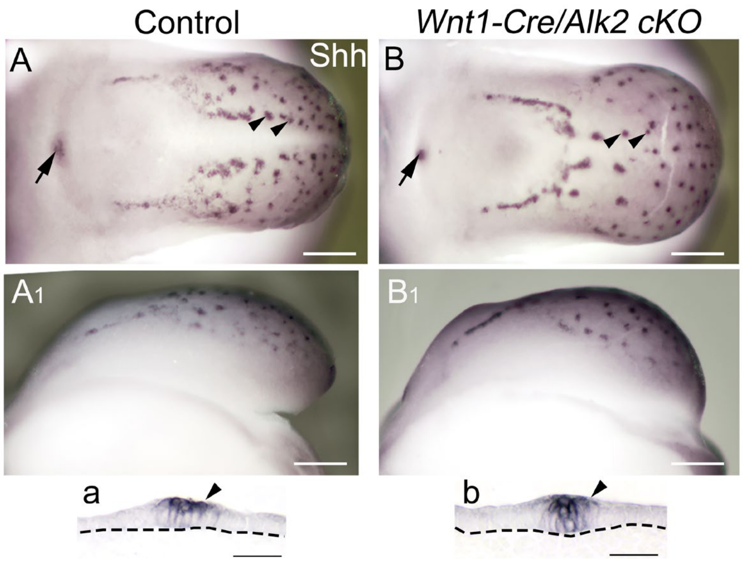

Fig. S1. Taste papillae develop in the E12.0 *Wnt1-Cre/Alk2 cKO* mice. Light microscopy images of E12.5 *Cre*$^-$*/Alk2*$^{fx/fx}$ (A, A_1) and *Wnt1-Cre/Alk2 cKO* (B, B_1) tongues that were immunostained for Shh. A-B: dorsal view; A_1-B_1: side view; a-b: sagittal tongue sections of *Cre*$^-$ */Alk2*$^{fx/fx}$ (a) and *Wnt1-Cre/Alk2 cKO* (b) mice. Dashed lines demarcate the tongue epithelium from the underlying mesenchyme. Arrowheads and arrows point to Shh$^+$ fungiform and circumvallate papilla placodes respectively. Scale bars: 200 μm in A-B, 25 μm in a-b.

Development • Supplementary information

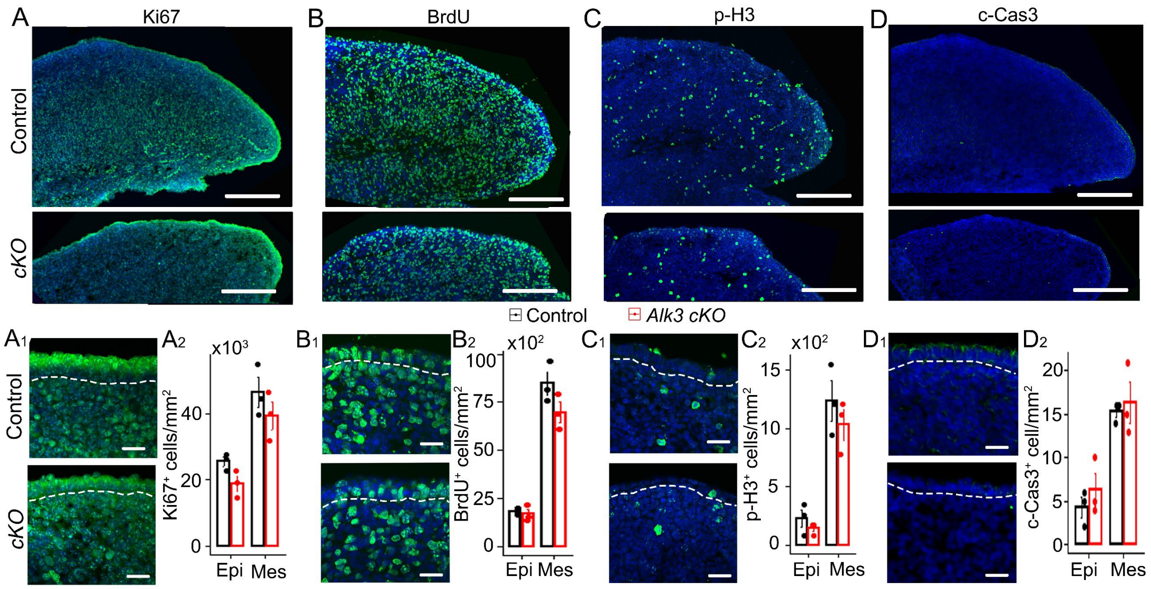

Fig. S2. Cell proliferation was unaltered and apoptotic cells were rarely seen in the E12.0 *Wnt1-Cre/Alk3 cKO* tongues. A-D: Single-plane laser scanning confocal images from E12.0 *Cre⁻/Alk3*$^{fx/fx}$ (Control) and *Wnt1-Cre/Alk3 cKO* (*Alk3 cKO*) tongue sections immunostained for cell proliferation markers Ki67 (pan, green in A), BrdU (S-phase, green in B), p-H3 (M-phase, green in C) or apoptosis marker cleaved-caspase 3 (c-Cas3, green in D). A_1- D_1: High-magnification images of the anterior tongue tip. Dashed lines demarcate the tongue epithelium from the underlying mesenchyme. Scale bars: 50 µm. A_2-D_2: Histograms (X±SD; n=3) to present the number of Ki67$^+$, BrdU$^+$, p-H3$^+$ and c-Cas3$^+$ cells per mm^2 in the epithelium (Epi) and mesenchyme (Mes) of *Cre⁻/Alk3*$^{x/fx}$ and *Wnt1-Cre/Alk3 cKO* tongues. No statistically significant differences were found in *Wnt1-Cre/Alk3 cKO* compared to the corresponding regions of *Cre⁻/ Alk3*$^{fx/fx}$ littermate control using two-way ANOVA followed by Fisher's LSD analyses.

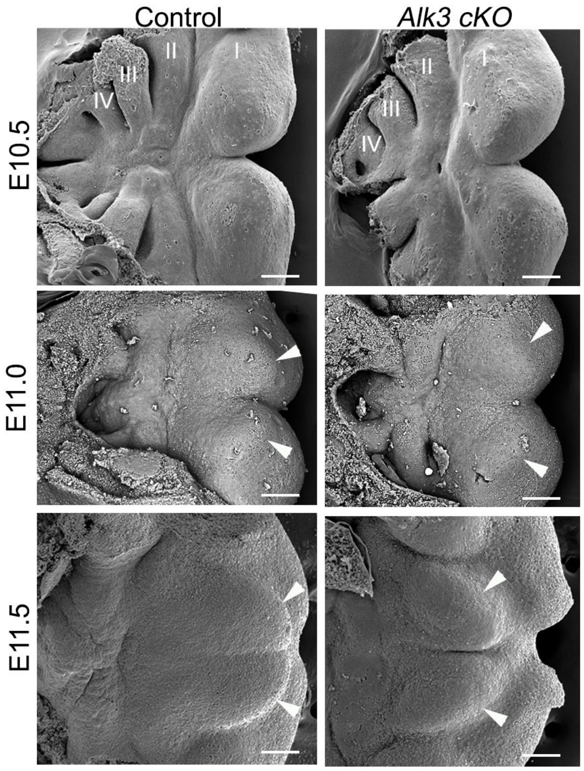

Fig. S3. *Wnt1-Cre/Alk3 cKO* mice do not depict an obvious phenotype of tongue development at early stages until E11.5. Scanning electron microscopy images of E10.5-E11.5 *Wnt1-Cre/Alk3 cKO* (*Alk3 cKO*) and *Cre*$^{-}$*/Alk3*$^{fx/fx}$ littermate (Control) mice. Arrowheads point to the lateral tongue swellings. The Roman numeral I-IV represents branchial arches 1-4. Scale bars: 100 μm.

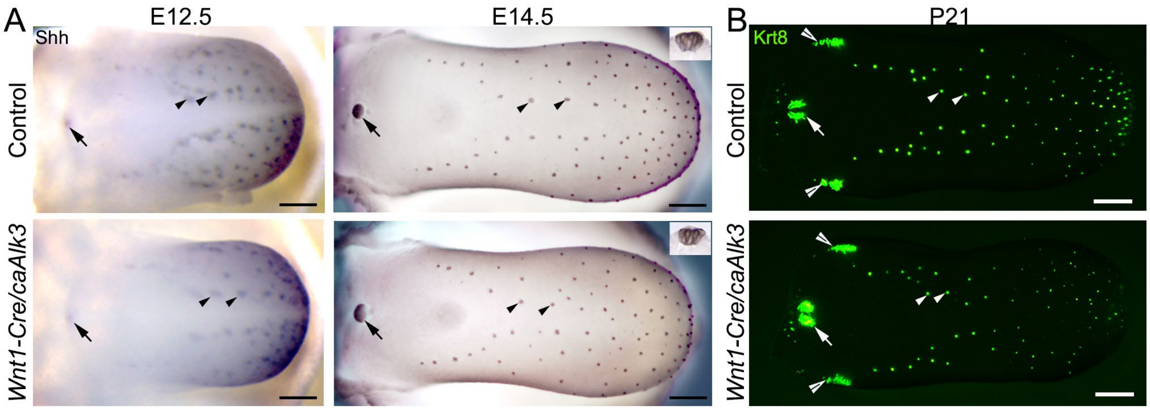

Fig. S4. Constitutive activation (ca) of *Alk3* in the tongue mesenchyme (*Wnt1-Cre*) does not alter the development of taste papillae and taste buds. **A**: Light microscopy images of E12.5 and E14.5 *Wnt1-Cre/caAlk3* and *Cre*$^{-}$ littermate (Control) tongues. Tongues were immunostained for taste papilla marker Shh (blue). Insets are light microscopy images of sagittal tongue sections. Scale bars: 200 µm. **B:** Stereomicroscopy images of postnatal day 21 (P21) control and *Wnt1-Cre/caAlk3* tongue epithelial sheets. Epithelial sheets were immunostained for pan-taste cell marker Krt8. Arrowheads, open arrowheads, and arrows point to fungiform, foliate, and circumvallate taste papillae/buds respectively. Scale bars: 500 µm.

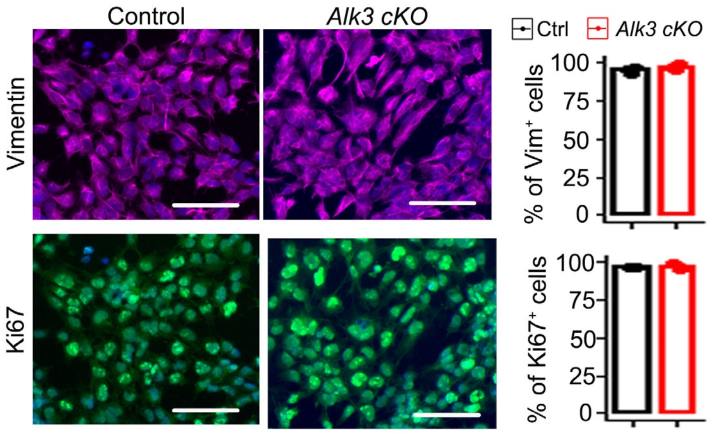

Fig. S5. The morphology and proliferation of tongue mesenchymal cells are unaltered in the *Wnt1-Cre/Alk3 cKO*. Single-plane laser scanning confocal images of cultured E11.5+3-day mesenchymal cells from *Cre⁻/Alk3$^{fx/fx}$* (Control) and *Wnt1-Cre/Alk3 cKO* (*Alk3 cKO*) tongues. Cells were immunostained for mesenchymal cell marker Vimentin (magenta) and Ki67 (green), and counterstained with nuclear marker DAPI (blue). Scale bars: 50 µm. Histograms (X±SD; n=3) on the right present the percentage of Vimentin$^+$ or Ki67$^+$ cells relative to the total number of cells (DAPI$^+$) in the cultures. No statistically significant differences were found in *Alk3 cKO* group compared to the *Cre⁻/Alk3$^{fx/fx}$* littermate control group using two-way ANOVA followed by Fisher's LSD analyses.

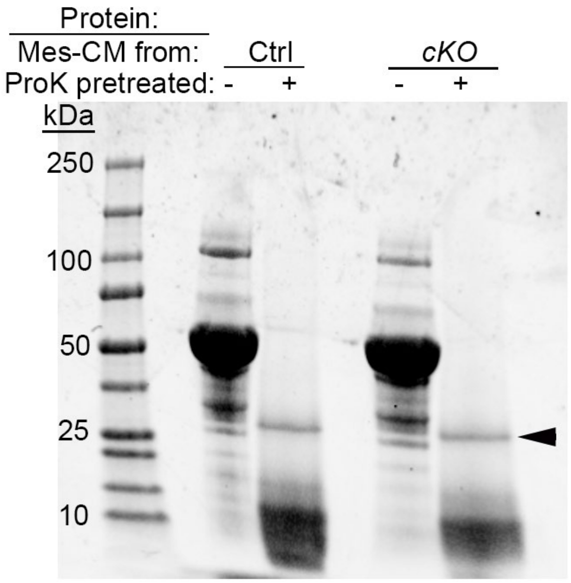

Fig. S6. Proteinase K pretreatment efficiently digests the proteins from mesenchyme-conditioned medium. A sodium dodecyl sulfate-polyacrylamide gel image to present the bands of proteins from mesenchyme-conditioned medium (Mes-CM) in *Cre⁻/Alk3$^{fx/fx}$* (Ctrl) and *Wnt1-Cre/Alk3 cKO* (*cKO*) mice without (-) or with (+) proteinase K (ProK) pretreatment. Protein bands are absent after ProK treatment. The arrowhead points to the band of ProK enzyme (28 kDa).

Development • Supplementary information

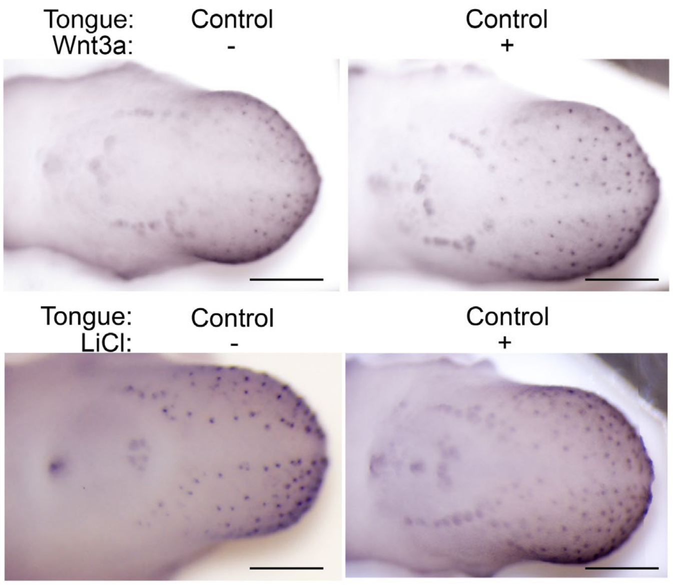

Fig. S7. Activation of Wnt/β-catenin signaling promotes taste papilla development in control tongue cultures. Representative light microscopy images of E12+2-day tongue cultures from *Cre⁻/Alk3*$^{fx/fx}$ (Control) mice. Tongue cultures were administered with 5 mM LiCl or 20% Wnt3a conditioned medium to activate Wnt/β-catenin signaling activity and immunostained for Shh (blue). Scale bars: 200 μm.

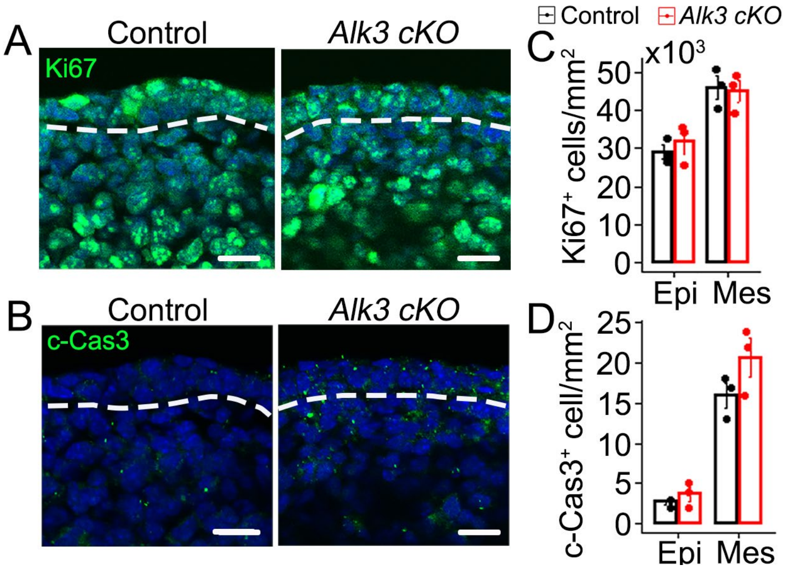

Fig. S8. *Sox10-Cre*-mediated mesenchyme-specific *Alk3 cKO* does not alter cell proliferation or apoptosis. **A-B**: Single-plane laser scanning confocal images from E12.0 *Cre⁻/Alk3$^{fx/fx}$* (Control) and *Sox10-Cre/Alk3 cKO* (*cKO*) tongue sections immunostained for Ki67 (green in A) or c-Cas 3 (green in B). These images are from sections of the anterior tongue tip at a high magnification. Dashed lines demarcate the tongue epithelium from the underlying mesenchyme. Scale bars: 50 µm. **C-D**: Histograms (X±SD; n=3) to present the number of Ki67$^+$ (C) and c-Cas3$^+$ (D) cells per mm^2 in the epithelium (Epi) and mesenchyme (Mes) of *Cre$^-$/Alk3$^{x/fx}$* and *Sox10-Cre/Alk3 cKO* tongues. No statistically significant differences were found in *Sox10-Cre/Alk3 cKO* compared to the corresponding regions of *Cre$^-$/Alk3$^{fx/fx}$* littermate control using two-way ANOVA followed by Fisher's LSD analyses.

Development • Supplementary information

Table S1. Bulk RNA-seq raw data

Click here to download Table S1

Table S2. *Alk3 cKO*-upregulated DEGs in the tongue epithelium

Click here to download Table S2

Table S3. *Alk3 cKO*-downregulated DEGs in the tongue epithelium

Click here to download Table S3

Table S4. Alk3 cKO-upregulated DEGs in the tongue mesenchyme

	baseMean	log2FoldChange	lfcSE	stat	pvalue	padj
Dlx4	9.67498757	-5.645098989	1.44481747	-3.90713644	9.34E-05	0.029513268
Th	31.6223061	-5.256039096	1.41699126	-3.70929536	0.0002078	0.049786985
Hmx1	17.3957028	-4.872117637	1.27452387	-3.82269627	0.000132	0.038440568
Pax1	178.231563	-4.161271938	0.39339614	-10.5778158	3.78E-26	5.61E-22
Zfp750	293.643283	-3.59409183	0.947968	-3.79136411	0.0001498	0.041984115
Rfx4	15.1794137	-3.166262199	0.73968224	-4.28057082	1.86E-05	0.008651965
4930583H1	44.7392234	-2.737971564	0.70394478	-3.88946926	0.0001005	0.031085141
Irx5	72.6574806	-2.663131998	0.57562042	-4.62654195	3.72E-06	0.003067941
Dlx3	262.048868	-2.398222585	0.46483413	-5.15930828	2.48E-07	0.000409031
Slc16a3	758.020507	-2.31396918	0.48281393	-4.79267274	1.65E-06	0.001764566
Hoxd8	21.2237455	-2.238953995	0.56620707	-3.95430244	7.68E-05	0.027805214
Cyp26a1	142.270332	-2.238427206	0.47760552	-4.68676992	2.78E-06	0.002424813
Bnc1	284.1885	-2.084455811	0.29251807	-7.12590449	1.03E-12	7.68E-09
Cpa2	52.3788309	-1.561003945	0.32679699	-4.77667779	1.78E-06	0.001764566
2310057J1	489.446013	-1.50340001	0.37568121	-4.00179721	6.29E-05	0.023939583
Pdk1	1544.41531	-1.499964387	0.34109806	-4.39745795	1.10E-05	0.005669573
Ak3l1	538.847441	-1.449210736	0.36838785	-3.9339265	8.36E-05	0.02887105
Ifitm1	128.030126	-1.300525246	0.34818854	-3.73511794	0.0001876	0.047971427
Lrrtm1	101.45058	-1.261564543	0.33671035	-3.74673522	0.0001792	0.04667984
Mpped1	77.5339633	-1.185961367	0.2776773	-4.27100579	1.95E-05	0.00875788
Bcl11b	1194.62512	-1.159653381	0.24599846	-4.71406763	2.43E-06	0.002253977
LOC10003	247.40801	-1.114621764	0.24405735	-4.56704858	4.95E-06	0.003498278
Ldha	7847.13191	-1.084098782	0.23562614	-4.60092755	4.21E-06	0.003123477
Ntf3	157.482127	-1.078220121	0.28573805	-3.7734566	0.000161	0.043505401
Rarb	745.4949	-1.036915921	0.22535463	-4.60126307	4.20E-06	0.003123477
LOC62000	68.6956604	-0.959725687	0.25510163	-3.7621307	0.0001685	0.044681171
Zfhx4	1213.30337	-0.950368831	0.14746712	-6.44461492	1.16E-10	5.74E-07
Pfkl	2450.00396	-0.935411452	0.24310327	-3.84779457	0.0001192	0.036125497
Nbl1	791.146509	-0.92738267	0.21065803	-4.40231343	1.07E-05	0.005669573
Tmem108	137.26252	-0.85345037	0.21238739	-4.0183665	5.86E-05	0.022904518
Lrba	854.776208	-0.771642602	0.18540997	-4.16181832	3.16E-05	0.013025348
Axin2	572.217737	-0.770883186	0.14915208	-5.16843745	2.36E-07	0.000409031
Pkm2	14062.8478	-0.711699741	0.12760109	-5.57753672	2.44E-08	7.25E-05
Tkt	2624.66879	-0.413732331	0.10785137	-3.83613405	0.000125	0.037125902

Table S5. Alk3 cKO-downregulated DEGs in the tongue mesenchyme

	baseMean	log2FoldChange	lfcSE	stat	pvalue	padj
Dhrs7c	9.434136664	3.05403681	0.778520976	3.922870292	8.75E-05	0.028871
Smpx	13.80177688	2.321840831	0.591107923	3.927947403	8.57E-05	0.028871
9430022F06Rik	22.50949872	2.242034677	0.510117382	4.395134833	1.11E-05	0.00567
Cryab	40.29638223	2.120543259	0.365643393	5.799484688	6.65E-09	2.47E-05
Sgcg	23.70174391	1.945034852	0.495248725	3.927389924	8.59E-05	0.028871
Ablim3	184.4415594	1.904032121	0.384450325	4.952608949	7.32E-07	0.001075
Actn2	288.9271672	1.838076133	0.463058177	3.969428089	7.20E-05	0.02675
Myh7	144.8773952	1.740132415	0.399853952	4.351920008	1.35E-05	0.006681
Itgb1bp2	59.62279828	1.724390722	0.349330679	4.936270485	7.96E-07	0.001075
Trim72	19.06103796	1.713045659	0.459113546	3.731202605	0.00019057	0.047971
Col9a3	364.099798	1.670415344	0.442694769	3.773289096	0.00016111	0.043505
Pgam2	53.91380221	1.46755117	0.340764843	4.306639016	1.66E-05	0.007941
Hrc	69.5961536	1.439169588	0.277894762	5.178829488	2.23E-07	0.000409
Col22a1	67.27273309	1.436296347	0.342303974	4.195967496	2.72E-05	0.01153
Pygm	53.84612814	1.319598144	0.275565815	4.788685942	1.68E-06	0.001765
Sytl2	188.8204938	1.25591813	0.329954448	3.806337935	0.00014104	0.040283
Myo5b	260.1754307	1.131097515	0.205804769	5.495973301	3.89E-08	9.62E-05
Wdr44	93.94052263	1.118714957	0.247597272	4.518284666	6.23E-06	0.004026
2310045A20Rik	96.77040443	1.095843978	0.279720448	3.917639862	8.94E-05	0.028871
Palmd	113.913406	1.09244044	0.25991481	4.203071146	2.63E-05	0.011502
Fzd8	200.2670596	1.049297693	0.23650907	4.436606561	9.14E-06	0.005429
Etv1	159.4297091	0.913944652	0.201944682	4.525717858	6.02E-06	0.004026
Pdzd2	355.3698363	0.900366552	0.200386009	4.493160752	7.02E-06	0.004343
Cplx2	917.8389655	0.887104219	0.217286884	4.082640438	4.45E-05	0.017873
Srl	561.7790758	0.722989508	0.194284484	3.721293096	0.00019821	0.048258
Myo6	522.2228977	0.705509839	0.16041636	4.397991816	1.09E-05	0.00567
Osbpl5	511.1545823	0.685476178	0.139885945	4.900250544	9.57E-07	0.001185
Peli2	466.5987111	0.663489922	0.178161187	3.724099138	0.00019601	0.048258

Table S6. Primary antibodies used for immunohistochemistry

Primary antibody	Source (catalog number, company)	Dilution
Rat anti-BrdU	MCA2060, Bio Rad, Hercules, CA	1:500
Rabbit anti-β-catenin	C2206, Sigma Aldrich, St Louis, MO	1:500
Rabbit anti-c-Cas3	#9661, Cell Signaling, Danvers, MA	1:500
Goat anti-E-cadherin	AF748, R&D Systems, Minneapolis, MN	1:1000
Sheep anti-FGF10	AF6224, R&D Systems, Minneapolis, MN	1:10,000
Goat anti-Follistatin	AF669, R&D Systems, Minneapolis, MN	1:10,000
Mouse anti-Gapdh	G8795, Sigma Aldrich	1:10,000
Rat anti-Krt8	TROMA-I, Developmental Studies Hybrydoma Bank, Iowa city, IA	1:1000
Sheep anti-Ki67	AF7649, R&D Systems, Minneapolis, MN	1:500
Goat anti-Noggin	AF719, R&D Systems, Minneapolis, MN	1:10,000
Rabbit anti-p-H3	#9701, Cell Signaling, Danvers, MA	1:500
Rabbit anti-Prox1	PA585552, Thermo Fisher Scientific, Waltham, MA	1:100
Rabbit anti-p-Smad1/5/8	#13820, Cell Signaling, Danvers, MA	1:500
Goat anti-Shh	AF464, R&D Systems, Minneapolis, MN	1:300
Chicken anti-Vimentin	AB5733, EMD Millipore, Burlington, MA	1: 500
