# Supplementary material for: Taste papilla cell differentiation requires the regulation of secretory protein production by ALK3-BMP signaling in the tongue mesenchyme
Source: Development. 2023 Sep 25;150(18):dev201838. doi: 10.1242/dev.201838 (PMC10560570; doi:10.1242/dev.201838)
Supplement: Supplementary file 1 [file develop-150-201838_review_history.pdf]

Taste papilla cell differentiation requires the regulation of secretory protein production by ALK3-BMP signaling in the tongue mesenchyme

Mohamed Ishan, Zhonghou Wang, Peng Zhao, Yao Yao, Steven L Stice, Lance Wells, Yuji Mishina and Hong-Xiang Liu

DOI: 10.1242/dev.201838

Editor: Patrick Tam

Review timeline

Original submission:	3 April 2023
Editorial decision:	10 May 2023
First revision received:	9 August 2023
Accepted:	1 September 2023

Original submission

First decision letter

MS ID#: DEVELOP/2023/201838

MS TITLE: Taste papilla cell differentiation requires tongue mesenchyme via ALK3-BMP signaling to regulate the production of secretory proteins

AUTHORS: Mohamed Ishan, Zhonghou Wang, Peng Zhao, Yao Yao, Steven L Stice, Lance Wells, Yuji Mishina, and Hongxiang Liu

I have now received all the referees' reports on the above manuscript, and have reached a decision. The referees' comments are appended below, or you can access them online: please go to BenchPress and click on the 'Manuscripts with Decisions' queue in the Author Area.

As you will see, the referees express considerable interest in your work, but have some significant criticisms and recommend a substantial revision of your manuscript before we can consider publication. If you are able to revise the manuscript along the lines suggested, which may involve further experiments, I will be happy receive a revised version of the manuscript. Your revised paper will be re-reviewed by one or more of the original referees, and acceptance of your manuscript will depend on your addressing satisfactorily the reviewers' major concerns. Please also note that Development will normally permit only one round of major revision. If it would be helpful, you are welcome to contact us to discuss your revision in greater detail. Please send us a point-by-point response indicating your plans for addressing the referee’s comments, and we will look over this and provide further guidance.

Please attend to all of the reviewers' comments and ensure that you clearly highlight all changes made in the revised manuscript. Please avoid using 'Tracked changes' in Word files as these are lost in PDF conversion. I should be grateful if you would also provide a point-by-point response detailing how you have dealt with the points raised by the reviewers in the 'Response to Reviewers' box. If you do not agree with any of their criticisms or suggestions please explain clearly why this is so.

© 2023. Published by The Company of Biologists under the terms of the Creative Commons Attribution License (https://creativecommons.org/licenses/by/4.0/).

Reviewer 1

Advance summary and potential significance to field

Taste papilla cell differentiation requires tongue mesenchyme via ALK3-BMP signaling to regulate the production of secretory proteins The study shows that during embryonic development, bone morphogenetic protein (BMP) signaling mediated by the ALK3 receptor in the mesenchyme of the tongue is necessary for taste papilla cell differentiation

Comments for the author

Taste papilla cell differentiation requires tongue mesenchyme via ALK3-BMP signaling to regulate the production of secretory proteins The study shows that during embryonic development, bone morphogenetic protein (BMP) signaling mediated by the ALK3 receptor in the mesenchyme of the tongue is necessary for taste papilla cell differentiation. Knockout of Alk3 in the mesenchyme resulted in the absence of taste papillae. The ALK3-BMP signaling controls the production of secretory proteins that promote taste cell differentiation. The study also reveals that mesenchymal ALK3-BMP signaling governs epithelial Wnt/β-catenin signaling in taste papilla cell differentiation. However few questions need to be answered before the paper is accepted for publication.
1. In Fig 1A and B, authors state that at E12.5 p-smad1/5/8+ cells are abundant in the mesenchyme of the tongue. However, based on the histogram of FPKM smad8 expression is almost zero. So, the Fig B expression maybe of only smad5 cells and that smad5 may be activated by non-canonical signaling pathway. Please explain your opinion on this comment.
2. The mesenchyme in the developing tongue is derived from neural crest cells during development. Sox10 is the most prominent marker or neural crest cells which is also mentioned by the author in the paper. Than what is the reason for using Wnt1 cre mice for the most experiments and excluding Sox10 cre mice?
3. During the tongue culture in Fig. 3, why was the whole tongue used instead of only the tongue epithelium when the aim of the experiment was to see the effect of mesenchyme from the cKO mice on the epithelium?
5. Page 7, Para 2, To define the tongue mesenchyme-derived …. , rephrase that sentence.
6. In Fig. 4. ProK was used to digest all the proteins, than how come the protein that promotes differentiation of taste cell papilla were maintained? In my opinion, since all the proteins are degraded there shouldn't be any papilla formation as both promoting and inhibitory proteins should be degraded.
7. Constitutive activation of Alk3 did not show any difference in taste papilla cells, than how come the administration of 10-100-kDa protein lead to increase in the number of taste papilla? This is a bit confusing to me.
8. In Fig. 5, to confirm that wnt/b-catenin pathway is downregulated, authors may perform the immunohistochemistry to show the localization of b-catenin either in the nucleus or cytoplasm in control and cKO group as only activated b-catenin will be expressed in the nucleus and therefore it would be a good figure to explain the downregulation of b-catenin in the WT and cKO.
9. Since the authors have the RNA seq and LC-MS data, it would be better if you can find the exact secretory factor responsible for communication between BMP and Wnt signaling during papilla development.
10. The inset diagrams in the figures are too small, please enlarge them. Also in the initial figure 2 mice strains are used, so add the strain name in the figures as its confusing to understand especially in the case of supplemental figure 3. In the same figure, there are no labels D,E and F only D1, D2 … but they are mentioned in figure legends. Check that.
11. Also include in the discussion, the possibility of truncated tongue observed after cKO.

Reviewer 2

Advance summary and potential significance to field

The subject of tongue development is not as well reported as many other aspects of craniofacial development and this paper should be welcomed. It very nicely demonstrates a role for BMP and WNT signalling during differentiation of the tongue epithelium using mouse models, organ culture and genomic analysis.

© 2023. Published by The Company of Biologists under the terms of the Creative Commons Attribution License (https://creativecommons.org/licenses/by/4.0/).

Comments for the author

This is a nice paper describing the role of BMP signalling via the ALK3 receptor in taste papilla differentiation – specifically through WNT/B-catenin activity. The experiments have been robustly conducted and I think this is an interesting story that should be published in Development. I think the narrative of the report and conclusions of some of the individual experiments could be better reported but overall – nice paper.

I am not sure how Fig A relates to Shh; Krt8 activity in the epithelium? (line 21 page 4). An image of this expression might be useful

Page 5 line 7 – ‘largely labels' is the wrong phrase here I think – these mice are being used to knock out gene function not label cells

BMP signalling is in the core of the tongue mesenchyme according to pSmad158 expression shown (in Fig 1B) with Alk3 pretty much everywhere (Fig 1D)? In the Wnt1-Cre/Alk3 KO pSmad158 is reduced in mesenchyme below the epithelium although not completely lost. There does not seem to be much discussion of this absence of BMP signal immediately below the epithelium but activity in the core? Is this because pSmad activity relates to cell origin? Is it only in CNCC cells? Presumably not, and if not – what is the relevance?

Too many paragraphs start with….to understand

Top of page 7 – this seems like a strange list of papers to illustrate tissue-tissue or cell-cell interactions?
The Rice paper relates to molecular signalling in the secondary palate.

The tongue co-culture experiments described on page 7 do not really tell me whether the interactions are indeed tissue-tissue or cell to cell? Am I missing something? The conclusion from the result described in Figure 4 should be more clearly articulated.

Reviewer 3

Advance summary and potential significance to field

Studies of the development of taste buds in the embryonic tongue have focused primarily on the role of epithelial signals. Here Ishan et al. have made the novel observation that genetic perturbation of Bmpr1a/Alk3 in the neural crest (NCC)-derived lingual mesenchyme causes loss of taste primordia in the epithelium, suggesting that signals downstream of BMP/ALK3 from the mesenchyme to the epithelium are required for taste bud development. This model is further supported by experimental results where tongues from wild type embryos are cultured in conditioned media from separately cultured control or mutant lingual mesenchyme; mutant media repressed formation of taste primordia, while WT media had no effect. The authors concluded that Bmpr1a/Alk3 is required to drive expression of signals that promote taste differentiation and/or repress signals that inhibit taste differentiation. They go on to show that in a fractionation assay, that mesenchymal proteins from mutants between 10-100kDa but not larger or smaller, are responsible for the repressive effect in tongue cultures. The authors then used bulk RNAseq to compare transcriptomes of epithelium and mesenchyme from WT and mutant tongues and identify signaling pathway genes that are altered in mutant v WT. Surprisingly, they find many more genes are affected in epithelium compared to mesenchyme.

Specifically, several Wnt/β-catenin genes are reduced, while a Wnt antagonist, Sostdc1, is upregulated, as are some BMP pathway genes. Shh target genes are not affected (nor is Shh itself in Suppl table 1 in contrast to expression data in figs 2-4), which was not addressed by the authors. Epithelial Wnt/β-catenin signaling has been shown by others to be required for taste placode formation and differentiation, so here the authors tested the hypothesis that reduced Wnt signaling downstream of mesenchymal Alk3 cKO led to loss of taste primordia by supplementing mutant

© 2023. Published by The Company of Biologists under the terms of the Creative Commons Attribution License (https://creativecommons.org/licenses/by/4.0/).

tongue cultures with LiCl or Wnt3a to activate the pathway. Lithium rescued the taste phenotype, while Wnt3a did not, suggesting Wnt ligand antagonists from mutant lingual epithelium and mesenchyme are blocking Wnt ligand and thereby repressing taste development. (Importantly, Wnt3a augmented taste development in control tongues.) The authors also showed that lithium rescued the inhibitory effect of conditioned medium from mutant mesenchyme on control tongues. In figure 6, the authors interrogated the mesenchyme bulk seq dataset from control vs mutant. Their GO term analysis indicated that exocytosis was the most highly downregulated process in mutant mesenchyme, while Wnt signaling genes were mostly comparable between mutants and WT, suggesting changes in mesenchymal Wnt were not responsible for the Alk3 cKO taste phenotype, but based on RNA expression do not identify potential candidate factors responsible. Finally, the authors compare LC-MS analysis of proteins released in the culture medium of WT vs control mesenchyme, identifying biological processes differentially enriched in each, but not potential candidates responsible. Moreover, known mesenchymal regulators of taste placode patterning, FGF10 and FST were also unaltered in mutant v control media.

Overall this is an interesting and novel set of findings but there are several issues that impact interpretation of results, and insight into potential mechanisms.

Comments for the author

Major comments:

1. In controls, taste bud primordia express Shh, among other markers. Using only Shh to identify nascent taste buds, however, the authors report that deletion of Alk3 in lingual NCC causes a “complete loss of taste papillae”. To make such a strong statement, the authors should assess expression of other genes/proteins expressed in taste placodes, including SOX2, PROX1, FOXA2 (Okubo et al. doi: 10.1101/gad.1457106, Nakayama et al. DOI: 10.1002/cne.21738 , Golden et al. doi: 10.7554/eLife.64013), and ascertain these are also absent in mutants compared to controls. Taste primordia are also evident in histological sections as epithelial thickenings, and the authors could use this approach as well to determine if taste placodes are absent.
   a. How do the authors reconcile the loss of Shh expression in tongues in Figs 2-4 with partly reduced transcript levels in mutant vs wt epithelium in suppl table 1?
2. The authors propose in the discussion that normal Alk3 function inhibits production of taste development inhibitors, which then are overexpressed/overproduced in mutants. Can they thus phenocopy the effect of genetic deletion with Alk3 inhibition in WT cultures?
3. Bmpr1a transcript levels are comparable in WT and Alk3 cKO mesenchyme in suppl table 1. Is this because Alk3 is also highly expressed in the tongue muscle derived from mesoderm and therefore masks reduction in NCC which is a smaller subset of the lingual mesenchyme? Along these lines, are mesenchymal cultures primarily NCC-derived mesenchyme, or do these also contain mesodermal muscle cells? And how comparable are these cultures to the composition of cells used for the RNAseq? This discrepancy may obscure the authors’ ability to identify candidates likely to underlie the mutant phenotype. Better characterization of mesenchyme cultures and isolation and sequencing of NCC-derived mesenchyme would improve chances that testable candidate factors/pathways will be identified.

Minor comments:

A. Fig 6A. add DEG lists for the Venn Diagram panel as another table in the suppl table 1 file.

First revision

<u>Author response to reviewers' comments</u>

We extend our sincere gratitude to all the reviewers for their valuable feedback. We have carefully addressed each of their comments and incorporated the necessary changes and explanations to enhance the clarity of the manuscript. The revisions in the manuscript and our responses to reviewers’ comments are marked in red for easy identification.

© 2023. Published by The Company of Biologists under the terms of the Creative Commons Attribution License (https://creativecommons.org/licenses/by/4.0/).

Reviewer 1 Advance Summary and Potential Significance to Field: Taste papilla cell differentiation requires tongue mesenchyme via ALK3-BMP signaling to regulate the production of secretory proteins The study shows that during embryonic development, bone morphogenetic protein (BMP) signaling mediated by the ALK3 receptor in the mesenchyme of the tongue is necessary for taste papilla cell differentiation

Reviewer 1 Comments for the Author:
Taste papilla cell differentiation requires tongue mesenchyme via ALK3-BMP signaling to regulate the production of secretory proteins The study shows that during embryonic development, bone morphogenetic protein (BMP) signaling mediated by the ALK3 receptor in the mesenchyme of the tongue is necessary for taste papilla cell differentiation. Knockout of Alk3 in the mesenchyme resulted in the absence of taste papillae. The ALK3-BMP signaling controls the production of secretory proteins that promote taste cell differentiation. The study also reveals that mesenchymal ALK3-BMP signaling governs epithelial Wnt/β-catenin signaling in taste papilla cell differentiation. However few questions need to be answered before the paper is accepted for publication.

1. In Fig 1A and B, authors state that at E12.5 p-smad1/5/8+ cells are abundant in the mesenchyme of the tongue. However, based on the histogram of FPKM smad8 expression is almost zero. So, the Fig B expression maybe of only smad5 cells and that smad5 may be activated by non-canonical signaling pathway. Please explain your opinion on this comment.
It is true that the FPKM value of Smad8 is lower than Smad1 and 5. However, the transcripts are detected consistently in the replicates (FPKM values in 3 tissue pooled x 3 replicates are 2.224769, 2.242641, and 2.361174). Our immunohistochemistry showed robust signals of p- Smad1/5/8; however, because the antibody recognizes phospholylated Smad1, 5 and 8, it is not possible to distinguish which one is present in the tongue mesenchyme. In the revised version, we have added data using BMP-Smad activation inhibitor Dorsomorphin to treat the cultured mesenchymal cells. The proteins from the conditioned medium of Dorsomorphin-treated mesenchymal cells mimicked *Alk3 cKO* papilla loss. Further, the previous reports (Liu et al., Genesis 2018; Yumoto et al., J Biol Chem 2013) showed, although not mentioned literally, that taste papillae form after the loss-of-function of non-canonical (pSmad1/5/8-independent) BMP signaling. Together, these data indicate that it is less likely that Smad5 is activated by non- canonical signaling pathway and activation of p-Smad1/5/8 through ALK3 kinase activity (canonical pathway) regulates the production of proteins and epithelial cell differentiation. Studies using the specific knockout of each factor (Smad1 or 5 or 8) for a comparison will be beneficial to defining which one(s) is playing a critical role. We have elaborated our discussion on this matter.

2. The mesenchyme in the developing tongue is derived from neural crest cells during development. Sox10 is the most prominent marker or neural crest cells which is also mentioned by the author in the paper. Than what is the reason for using Wnt1 cre mice for the most experiments and excluding Sox10 cre mice?
Our original studies (phenotypic analyses and RNA-Seq) used *Wnt1-Cre* mostly. To be consistent and comparable with the data from those analyses, we continued using Wnt1-Cre for further mechanistic studies. In this revised version, we added new data (new Fig. 6) from *Sox10-Cre/Alk3 cKO* which are consistent with the important findings from *Wnt1-Cre/Alk3 cKO*.

3. During the tongue culture in Fig. 3, why was the whole tongue used instead of only the tongue epithelium when the aim of the experiment was to see the effect of mesenchyme from the cKO mice on the epithelium?
We have tested the cultures using separated tongue epithelium at this early stage. However, epithelial cells are undergoing rapid differentiation and enzyme digestion for the separation of the epithelium affected cell survival and disrupted the cell differentiation. Therefore, to keep the integrity of tongue epithelium, we used the whole tongue organ for co-cultures with the cKO mesenchyme. In the revised manuscript, we have added descriptions to explain this in both Methods and Results.

There was no question 4 from the Reviewer 1.

5. Page 7, Para 2, To define the tongue mesenchyme-derived …., rephrase that sentence.
In the revised manuscript we have rephrased the term as “mesenchyme-produced proteins” to be

© 2023. Published by The Company of Biologists under the terms of the Creative Commons Attribution License (https://creativecommons.org/licenses/by/4.0/).

clear.

6. In Fig. 4. ProK was used to digest all the proteins, than how come the protein that promotes differentiation of taste cell papilla were maintained? In my opinion, since all the proteins are degraded there shouldn't be any papilla formation as both promoting and inhibitory proteins should be degraded.
We apologize for the confusion. In our experiment, ProK was used to digest the extracted proteins from *Alk3 cKO* conditioned medium as a pre-treatment. Following the digestion, ProK was inactivated before adding the digested protein products into the culture medium. Therefore, there was no active ProK in the wild-type tongue cultures and there should be no effect on the endogenous protein levels in the WT tongue itself; thus, in the wild type tongue cultures, the epithelium and mesenchyme were intact. As a result, taste papillae developed without the inhibition by *Alk3 cKO* mesenchyme-produced inhibitors. We have added descriptions for improved clarity.

7. Constitutive activation of Alk3 did not show any difference in taste papilla cells, than how come the administration of 10-100-kDa protein lead to increase in the number of taste papilla? This is a bit confusing to me.
The *ex vivo* administration of 10-100-kDa proteins represents an addition of a high dose of isolated protein fraction that contain active “promotants”. *In vivo* constitutive activation of *Alk3* is likely not enhancing the protein production to this level. We have added this explanation for improved clarity.

8. In Fig. 5, to confirm that wnt/b-catenin pathway is downregulated, authors may perform the immunohistochemistry to show the localization of b-catenin, either in the nucleus or cytoplasm in control and cKO group as only activated b-catenin will be expressed in the nucleus and therefore it would be a good figure to explain the downregulation of b-catenin in the WT and cKO.
In the revised version of the manuscript, we have added data to show ß-catenin immunosignals presented in Figure 5C. The new data illustrates that in the control group, ß-catenin is localized in both the cell nucleus and membrane, whereas in the *Alk3 cKO* group, ß-catenin was predominantly observed in the epithelial cell membrane.

9. Since the authors have the RNA seq and LC-MS data, it would be better if you can find the exact secretory factor responsible for communication between BMP and Wnt signaling during papilla development.
We also hoped to efficiently identify the exact secretory factors responsible for communication between BMP and Wnt signaling during papilla development. However, after having invested tremendous efforts, we realized that it is much more challenging than we thought. It was surprising to us that the differentially expressed genes from RNA-Seq and proteins from LC-MS data did not overlap and that the LC-MS data provides a long list, which makes the identification difficult. In this article, we present and have discussed the results. However, identifying the promising proteins individually is out of the scope of this study.

10. The inset diagrams in the figures are too small, please enlarge them. Also, in the initial figure 2 mice strains are used, so add the strain name in the figures as its confusing to understand especially in the case of supplemental figure 3. In the same figure, there are no labels D,E and F only D1, D2 … but they are mentioned in figure legends. Check that.
In the revised manuscript, we have reorganized our data for clarity. Fig. 2 has been significantly changed. The inset has been removed, and the relevant data is now presented as a separate panel (Fig. 2G, H) with a larger surrounding region for better context. To improve clarity, Fig. 2 includes data from one strain (*Wnt1-Cre/Alk3 cKO*). Furthermore, we present a separate figure (Fig. 6) for the key findings using *Sox10-Cre/Alk3 cKO*. Additionally, we revised the supplemental figure 3 (now supplemental figure 2 in the revised version) to include only the *Wnt1-Cre/Alk3 cKO* strain data. The data from *Sox10-Cre/Alk3 cKO* strain are presented as Supplemental Figure 8. In Supplemental Figure 2, we maintained consistent labeling, using panels A-D for low magnification images and panels A1-D1 for high magnification images, along with corresponding histograms in panels A2-D2.

11. Also include in the discussion, the possibility of truncated tongue observed after cKO.
In the revised manuscript we have included a separate section to discuss the growth of tongue and

© 2023. Published by The Company of Biologists under the terms of the Creative Commons Attribution License (https://creativecommons.org/licenses/by/4.0/).

mandible and the possibility of having a truncated tongue. Briefly, although the development of both tongue and mandible is interconnected and coordinated, their outgrowth is regulated by different molecular signaling pathways under different mechanisms.

Reviewer 2 Advance Summary and Potential Significance to Field:
The subject of tongue development is not as well reported as many other aspects of craniofacial development and this paper should be welcomed. It very nicely demonstrates a role for BMP and WNT signalling during differentiation of the tongue epithelium using mouse models, organ culture and genomic analysis.

Reviewer 2 Comments for the Author:
This is a nice paper describing the role of BMP signalling via the ALK3 receptor in taste papilla differentiation - specifically through WNT/B-catenin activity. The experiments have been robustly conducted and I think this is an interesting story that should be published in Development. I think the narrative of the report and conclusions of some of the individual experiments could be better reported but overall - nice paper.

1. I am not sure how Fig A relates to Shh; Krt8 activity in the epithelium? (line 21 page 4). An image of this expression might be useful
In the revised manuscript, we have removed Krt8 and included a more relevant marker Prox1. New data of Prox1 immunosignals which serve as another taste papilla cell marker have been added (Fig. 2I, J). These experiments were performed to provide further confirmation regarding the absence of high Shh-expressing taste papillae in the *Alk3 cKO*.

2. Page 5 line 7 - 'largely labels' is the wrong phrase here I think - these mice are being used to knock out gene function not label cells
We have changed the word "largely labels" to "which mark the NC cell lineage in the tongue mesenchyme extensively".

3. BMP signalling is in the core of the tongue mesenchyme according to pSmad158 expression shown (in Fig 1B) with Alk3 pretty much everywhere (Fig 1D)? In the Wnt1-Cre/Alk3 KO pSmad158 is reduced in mesenchyme below the epithelium although not completely lost. There does not seem to be much discussion of this absence of BMP signal immediately below the epithelium but activity in the core? Is this because pSmad activity relates to cell origin? Is it only in CNCC cells? Presumably not, and if not - what is the relevance?
In the revised manuscript, we have elaborated the discussions regarding the possibility of observing significantly reduced *Alk3* signals in the mesenchymal layer underneath the epithelium while signals remained in the deep layer of mesenchyme. During the early stages of tongue development, cranial neural crest (CNC)-derived cells populate the tongue mesenchyme and then non-NC-derived myoprogenitors migrate into the tongue. The migration of myoprogenitors emerges at the core of the deep-layer mesenchyme and spreads throughout the tongue while leaving the mesenchyme adjacent to the epithelium (future lamina propria) as a dense population of NC-derived cells. In this study, *Alk3* was specifically knocked out in the NC-derived mesenchyme using *Wnt1-Cre* and *Sox10-Cre*. Therefore, *Alk3 cKO* caused the absence of *Alk3* mainly in the mesenchyme closer to epithelium. We have added the explanation in the discussion.

4. Too many paragraphs start with….to understand
We have changed the wording to avoid repetitions.

5. Top of page 7 - this seems like a strange list of papers to illustrate tissue-tissue or cell-cell interactions? The Rice paper relates to molecular signaling in the secondary palate.
We have removed the unnecessary references and updated the reference list accordingly.

6. The tongue co-culture experiments described on page 7 do not really tell me whether the interactions are indeed tissue-tissue or cell to cell? Am I missing something? The conclusion from the result described in Figure 4 should be more clearly articulated.
In the revised manuscript, we have revised the subtitle and context for a clearer explanation and logic.

© 2023. Published by The Company of Biologists under the terms of the Creative Commons Attribution License (https://creativecommons.org/licenses/by/4.0/).

Reviewer 3 Advance Summary and Potential Significance to Field:

Studies of the development of taste buds in the embryonic tongue have focused primarily on the role of epithelial signals. Here Ishan et al. have made the novel observation that genetic perturbation of Bmpr1a/Alk3 in the neural crest (NCC)-derived lingual mesenchyme causes loss of taste primordia in the epithelium, suggesting that signals downstream of BMP/ALK3 from the mesenchyme to the epithelium are required for taste bud development. This model is further supported by experimental results where tongues from wild type embryos are cultured in conditioned media from separately cultured control or mutant lingual mesenchyme; mutant media repressed formation of taste primordia, while WT media had no effect. The authors concluded that Bmpr1a/Alk3 is required to drive expression of signals that promote taste differentiation and/or repress signals that inhibit taste differentiation. They go on to show that in a fractionation assay, that mesenchymal proteins from mutants between 10-100kDa but not larger or smaller, are responsible for the repressive effect in tongue cultures. The authors then used bulk RNAseq to compare transcriptomes of epithelium and mesenchyme from WT and mutant tongues and identify signaling pathway genes that are altered in mutant v WT.

Surprisingly, they find many more genes are affected in epithelium compared to mesenchyme. Specifically, several Wnt/ß-catenin genes are reduced, while a Wnt antagonist, Sostdc1, is upregulated, as are some BMP pathway genes. Shh target genes are not affected (nor is Shh itself in Suppl table 1 in contrast to expression data in figs 2-4), which was not addressed by the authors. Epithelial Wnt/ß-catenin signaling has been shown by others to be required for taste placode formation and differentiation, so here the authors tested the hypothesis that reduced Wnt signaling downstream of mesenchymal Alk3 cKO led to loss of taste primordia by supplementing mutant tongue cultures with LiCl or Wnt3a to activate the pathway. Lithium rescued the taste phenotype, while Wnt3a did not, suggesting Wnt ligand antagonists from mutant lingual epithelium and mesenchyme are blocking Wnt ligand and thereby repressing taste development. (Importantly, Wnt3a augmented taste development in control tongues.) The authors also showed that lithium rescued the inhibitory effect of conditioned medium from mutant mesenchyme on control tongues. In figure 6, the authors interrogated the mesenchyme bulk seq dataset from control vs mutant. Their GO term analysis indicated that exocytosis was the most highly downregulated process in mutant mesenchyme, while Wnt signaling genes were mostly comparable between mutants and WT, suggesting changes in mesenchymal Wnt were not responsible for the Alk3 cKO taste phenotype, but based on RNA expression do not identify potential candidate factors responsible. Finally, the authors compare LC-MS analysis of proteins released in the culture medium of WT vs control mesenchyme, identifying biological processes differentially enriched in each, but not potential candidates responsible. Moreover, known mesenchymal regulators of taste placode patterning, FGF10 and FST were also unaltered in mutant v control media.

Overall this is an interesting and novel set of findings but there are several issues that impact interpretation of results, and insight into potential mechanisms.

Reviewer 3 Comments for the Author:

Major comments:

1. In controls, taste bud primordia express Shh, among other markers. Using only Shh to identify nascent taste buds, however, the authors report that deletion of Alk3 in lingual NCC causes a “complete loss of taste papillae”. To make such a strong statement, the authors should assess expression of other genes/proteins expressed in taste placodes, including SOX2, PROX1, FOXA2 (Okubo et al. doi: 10.1101/gad.1457106, Nakayama et al. DOI: 10.1002/cne.21738 , Golden et al. doi: 10.7554/eLife.64013), and ascertain these are also absent in mutants compared to controls. Taste primordia are also evident in histological sections as epithelial thickenings, and the authors could use this approach as well to determine if taste placodes are absent.

It has been well documented that intense Shh immunosignals serve as a reliable marker for development taste papillae. Following the reviewer's suggestion, we have double-checked the serial sections of control and *Alk3 cKO* tongues that were immunostained for Shh. The epithelial thickening was consistently marked by Shh immunosignals and solely observed in the control tongue epithelium but not in the *Alk3 cKO*. Moreover, we conducted a new set of experiments using Prox1 to confirm the absence of taste papillae in the *Alk3 cKO*. The updated data are presented as Fig. 2G-H and Fig. 2I-J to illustrate the presence of Shh^{high} and Prox1+ epithelial thickening in control

© 2023. Published by The Company of Biologists under the terms of the Creative Commons Attribution License (https://creativecommons.org/licenses/by/4.0/).

and lack of the signals in *Alk3 cKO* tongue epithelium. We have revised the text in results accordingly to support our findings and improve the clarity.

2. How do the authors reconcile the loss of Shh expression in tongues in Figs 2-4 with partly reduced transcript levels in mutant vs wt epithelium in suppl table 1?
The previous publication has reported that the high level of Shh immunosignals in taste papillae and low levels of Shh immunosignals in the papilla-free regions including the interpapillary space and intermolar eminence (Liu et al., Dev Biol 2004). In this study, we used the high Shh protein expression as well as epithelial thickening and Prox1 expression as markers of developing taste papillae. The loss of high Shh-expressing taste papillae does not contradict the partly reduced transcript levels in mutant vs WT tongue epithelium since the low-level Shh expression is present in the papilla-free epithelium of *Alk3 cKO*. We have edited the term as Shh^{high} taste papilla and Shh^{low} inter-papilla cells.

3. The authors propose in the discussion that normal Alk3 function inhibits production of taste development inhibitors, which then are overexpressed/overproduced in mutants. Can they thus phenocopy the effect of genetic deletion with Alk3 inhibition in WT cultures?
We agree with the reviewer that it would be nice to phenocopy the effect of genetic deletion with Alk3 inhibition in WT tongue cultures. According to the suggestion, we have added new data using Dorsomorphin, an inhibitor of BMP-Smad activation, to treat the cultured WT mesenchymal cells. Using the proteins from Dorsomorphin-treated mesenchyme-conditioned medium, we mimicked the absence of taste papillae observed in the *Alk3 cKO* mice. The new results are presented in Fig 4C-D.

4. Bmpr1a transcript levels are comparable in WT and Alk3 cKO mesenchyme in suppl table 1. Is this because Alk3 is also highly expressed in the tongue muscle derived from mesoderm and therefore masks reduction in NCC which is a smaller subset of the lingual mesenchyme? Thanks for a great point! We agree with the reviewer that Alk3 is also highly expressed in the myoprogenitors derived from mesoderm (shown by the broad distribution of in situ hybridization signals) and therefore masks Alk3 reduction in NCC which is a subset of the lingual mesenchyme. We have added a paragraph to discuss this matter.

5. Along these lines, are mesenchymal cultures primarily NCC-derived mesenchyme, or do these also contain mesodermal muscle cells?
At the time (E11.5) when the tissue was collected for the mesenchymal cell cultures, very few myoprogenitors migrated into the tongue (Han et al, Development 2012). Therefore, the cultured cells are primarily, if not all, from neural crest-derived mesenchyme. We have added a description in the Materials and Methods to describe how the tissue collection was done for mesenchymal cell cultures.

6. And how comparable are these cultures to the composition of cells used for the RNAseq? This discrepancy may obscure the authors' ability to identify candidates likely to underlie the mutant phenotype. Better characterization of mesenchyme cultures and isolation and sequencing of NCC-derived mesenchyme would improve chances that testable candidate factors/pathways will be identified.
We admit that the composition of mesenchymal cells used for RNA-Seq are not pure NC- derived cells. Based on the distribution of Wnt1-Cre (published) and Sox10-Cre (Fig. 6A) labeled cells, we are sure that at E12.0 when the tongue mesenchyme is populated primarily by NC-derived cells. A population of, but not many, non-NC-derived myoprogenitors are present in the deep layer of the tongue mesenchyme (Han et al, Development 2012). For the purpose of identifying mesenchyme-produced individual proteins that mediate mesenchymal-epithelial interactions, we will take the reviewer's suggestions into consideration about how to better characterize the isolation and sequencing of NCC-derived mesenchyme.

Minor comments:
7. Fig 6A. add DEG lists for the Venn Diagram panel as another table in the suppl table 1 file.
We have provided the DEG list as supplemental tables 2-5.

© 2023. Published by The Company of Biologists under the terms of the Creative Commons Attribution License (https://creativecommons.org/licenses/by/4.0/).

Second decision letter

MS ID#: DEVELOP/2023/201838

MS TITLE: Taste papilla cell differentiation requires tongue mesenchyme via ALK3-BMP signaling to regulate the production of secretory proteins.

AUTHORS: Mohamed Ishan, Zhonghou Wang, Peng Zhao, Yao Yao, Steven L Stice, Lance Wells, Yuji Mishina, and Hongxiang Liu
ARTICLE TYPE: Research Article

I am happy to tell you that your manuscript has been accepted for publication in Development, pending our standard ethics checks.

Reviewer 1

Advance summary and potential significance to field

The study shows that during embryonic development, bone morphogenetic protein (BMP) signaling mediated by the ALK3 receptor in the mesenchyme of the tongue is necessary for taste papilla cell differentiation. This is an interesting paper as studies in taste bud and taste papilla development are still not very well reported and some of the aspects still remain unknown. Therefore this study shows a new findings of requirement of BMP signaling in taste papilla development.

Comments for the author

The authors have fairly incorporated all the comments in their revised manuscript and hence No further revision needed.

Reviewer 2

Advance summary and potential significance to field

This paper describes the role of BMP signalling mediated through the ALK3 receptor in the tongue mesenchyme in regulating Wnt/β-catenin activity in the epithelium and taste papilla differentiation. The demonstration of a requirement for tongue mesenchyme in taste papilla cell differentiation is an important and interesting observation.

Comments for the author

I am happy with the changes that have been made in relation to my original suggestions.

© 2023. Published by The Company of Biologists under the terms of the Creative Commons Attribution License (https://creativecommons.org/licenses/by/4.0/).
